# Effect of (H_2_O)_*n*_ (*n* = 0–3, 13) on the NH_3_ + OH reaction in the gas and liquid phases†

**DOI:** 10.1039/d2ra04931g

**Published:** 2022-09-30

**Authors:** Yujie Zhou, Qi Cao, Yu Yang, Dandan Ma, Quan Zhu, Jianyi Ma

**Affiliations:** Institute of Atomic and Molecular Physics, Sichuan University Chengdu 610065 China majianyi81@163.com; Reactor Operation and Application Research Sub-Institute, Nuclear Power Institute of China Chengdu 610041 China; School of Chemical Engineering, Sichuan University Chengdu 610065 PR China; Engineering Research Center of Combustion and Cooling for Aerospace Power, Ministry of Education, Sichuan University Chengdu 610065 PR China

## Abstract

We studied the effect of water clusters on the NH_3_ + OH reaction at both the DFT and CCSD(T) levels. The calculated rate constants for the pure reaction are 2.07 × 10^−13^ and 1.35 × 10^−13^ cm^3^ molecule^−1^ s^−1^ in the gas and liquid phases, respectively, and the gas-phase rate constants are consistent with the corresponding experimental result (1.70 × 10^−13^ cm^3^ molecule^−1^ s^−1^), while the liquid-phase rate constants are slightly smaller than the experimental value (5.84 × 10^−12^ cm^3^ molecule^−1^ s^−1^). In the gas phase, the presence of (H_2_O)_*n*_ (*n* = 1–3) decreases the rate constant compared to the pure NH_3_ + OH reaction, and these results are in agreement with many reported H_2_O-catalyzed reactions. For the liquid phase reaction, compared with the case of *n* = 0–3, when the size of the water molecule cluster surrounding the OH radical is *n* = 13, the rate constant of the title reaction increases. Our study also shows that proton transfer is also a factor which accelerates the liquid phase NH_3_ + OH reaction.

## Abstract 2

We used DFT and CCSD(T) methods with PCM solvation model to study the water cluster effect and solvation effect on the NH_3_ + OH reaction.

## Graphical abstract

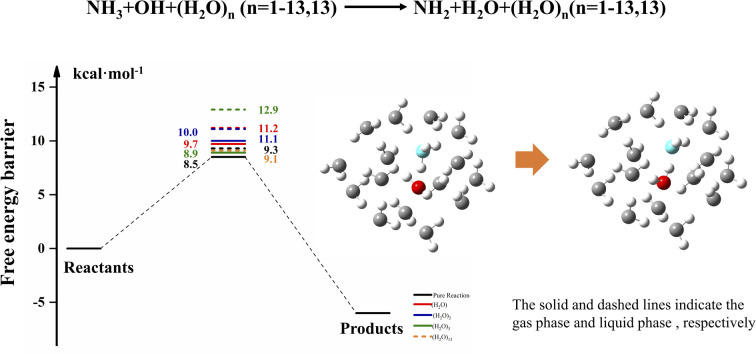

## 1. Introduction

Ammonia is the third most abundant nitrogen species in the terrestrial atmosphere and plays a highly critical role in both homogeneous and nonhomogeneous atmospheric reactions. Moreover, as the main alkaline gas in the atmosphere, ammonia can neutralize the acid in the atmosphere and can be oxidized to reduce the acidity of water.^1–4^ Currently, ammonia can be used as a new fuel instead of traditional fuels, and its combustion does not produce carbon dioxide, the products are water and nitrogen oxides.^5,6^ Hydroxyl radicals are widely present in water, the atmosphere and galactic space, and they serve as an extremely powerful oxidizing agent that can easily take hydrogen atoms from other molecules to form water molecules.^7–9^ The reaction of ammonia with hydroxyl radicals is a key step in the combustion mechanism of fossil fuels and is extremely important in the combustion process. As a typical hydrogen absorption reaction, the reaction of ammonia with hydroxyl radicals represents a decisive step in the oxidation of ammonia; therefore, this reaction has been studied extensively.^1,3,4,10–15^ On the other hand, the decomposition of coolant by strong radiation in the one-loop system produces strong oxidation products, which are an important cause of corrosion of structural materials and equipment. Ammonia is added to a water-cooled nuclear reactor to prevent oxygen production from radiolysis. The first step in ammonia decomposition is to react with the hydroxyl radicals produced by water irradiation. Therefore, the title reaction is extremely important in the ammonia radiolysis reaction in an aqueous phase environment.^16,17^

There are several studies of the NH_3_ + OH reaction both experimentally and theoretically. Experimentally, Perry^18^ determined the rate constant in the temperature range of 297–427 K and derived a rate constant of (1.64 ± 0.16) × 10^−13^ cm^3^ molecule^−1^ s^−1^ at room temperature. Silver^19^ then studied the rate constants in the temperature range from 294 to 1075 K, which gave the rate constants for the reaction at room temperature and extended the temperature range. Stephens^20^ also determined an absolute rate constant of 1.7 × 10^−13^ cm^3^ molecule^−1^ s^−1^ at room temperature by using the resonance fluorescence method. Wei-Guang Diau^4^ measured a rate constant of (1.47 ± 0.07) × 10^−13^ cm^3^ molecule^−1^ s^−1^ at 297 K by reaction dynamics. Hickel^17^ studied the reaction of hydroxyl radicals with ammonia in aqueous solutions by pulsed radiation analysis in the temperature range of 20–200 °C and measured a rate constant of 5.84 × 10^−12^ cm^3^ molecule^−1^ s^−1^ at room temperature. Theoretically, both Espinosa-Garcia and Giménez^1,11^ performed structural optimization and frequency calculations for reactants, transition states and reaction products. Espinosa-Garcia^21^ also calculated rate constants in the temperature range of 200–4000 K using variational transition-state theory. Giménez^1^ obtained enthalpy barriers of the NH_3_ + OH reaction of 5.12 kcal mol^−1^. In addition, Monge-Palacios^14^ based their higher-order *ab initio* calculations on the established full-dimensional analytical potential energy surface for the gas-phase reaction and performed a kinetic study, where their results well reproduced the experimental values of the forward rate constants. In a recent study, Zhang^22^ investigated the effect of water and ammonia as catalysts on the reaction rate constant at the CCSD(T)-F12a/cc-pVDZ-F12/M06-2X/6-311+G(2d,2p) level under gas-phase conditions. In Table 1, we list some of the previous work on the NH_3_ + OH reaction, both experimentally and theoretically. However, the solvation effect of the NH_3_ + OH reaction and how to calculate the NH_3_ + OH liquid phase rate constant is still a problem. As OH and NH_3_ are typical polar molecules, the solvation free energy changes greatly in the reaction process in water, a solvent with great polarity. Therefore, the influence of the solvation effect on the reaction needs to be considered for the NH_3_ + OH reaction in the liquid phase. In previous studies, many scholars have studied the solvation effect by adding solvent molecules and have achieved good results.^23–25^ Therefore, the same idea is used in this paper to study the effect of water clusters on the NH_3_ + OH reaction. The difference is that the former studied the spectral behavior by adding water molecules; we not only consider the solvation effect of water clusters but also the implicit solvent model and the proton transfer behavior, so we emphasize on the kinetic behavior.

Experimental and theoretical correlation work done on NH_3_ + OH reaction

**Table:** 

Experimental	
Ref. 4	The flash photolysis/laser-induced fluorescence technique was used to study the rate in 273–433 K
Ref. 17	The rate constants in the range of 293–473 K were studied by pulse radiolysis method in liquid phase
Ref. 18	The rate constants in the range of 297–427 K were studied by a flash photolysis-resonance fluorescence technique
Ref. 19	The rate constants in the range of 294–1075 K were obtained by high temperature fast flow reactor
Ref. 20	Study rate constants in 297–364 K by discharge flow technique with resonance fluorescence detection of OH

**Table:** 

Theoretical	
Ref. 2	Activation energies and rate constants in the 300–2500 K were calculated at the M06-2X/aug-ccpvqz and CCSD(T)/6-311++G(3df, 3pd) levels
Ref. 11	The energy variations was calculated at the QCI/6-311+G(2df,p) level
Ref. 14	Develop a full-dimensional analytical potential energy surface and calculate the rate constant in 200–200 K
Ref. 22	Study the effect of the addition of single water molecules on the rate at CCSD(T)-F12a/ccpVDZ-F12//M06-2X/6-311+G(2d,2p) level

In this work, we perform *ab initio* calculations for the H_2_O-catalyzed OH + NH_3_ reaction at the CCSD(T)-F12a/cc-pVDZ-F12//M06-2X/6-311+G(2d,2p) and M06-2X/6-311+G(2d,2p) levels for the gas phase and liquid phase, respectively. For each size water cluster, we further calculated the structure, reaction energy barrier and reaction rate of various conformations in gas and liquid phases. The corresponding rate constants are calculated by using transition state theory. In this case, we used the polarizable-continuum model (PCM)^26–28^ model from the implicit solvent model to consider the liquid-phase NH_3_ + OH reaction. And we calculate the solute free energy and solvation free energy together by the self-consistent solvation field.

## 2. Calculational method

The electronic structure calculation is performed by using Gaussian 09,^29^ MOLPRO^30^ program package. The structure optimization and energy calculation of all reactants, intermediates, transition states and products are performed using density functional theory (DFT) at the M06-2X/6-311+G(2d,2p) level^31,32^ in the liquid phase, which uses an implicit solvent model,^28,33^ the implicit solvent model used in this paper is the PCM model. In the gas phase, the optimized geometries are at the M06-2X/6-311+G(2d,2p) level, and the energy calculation is at the CCSD(T)-F12a/cc-pVDZ-F12 level.^34^ The accuracy of the M06-2X method in the reaction of ammonia and hydroxyl radicals has been confirmed in the past.^2^ At the same level, the minimum energy path (MEP) is also obtained through the intrinsic reaction coordinate (IRC)^35,36^ theory.

In the aqueous environment, water clusters with different sizes and conformations have a great probability of participating in the reaction. The processes for the formation of NH_3_ + OH + (H_2_O)_*n*_ → NH_2_ + H_2_O + (H_2_O)_*n*_ are divided into three main steps:^22,37,38^
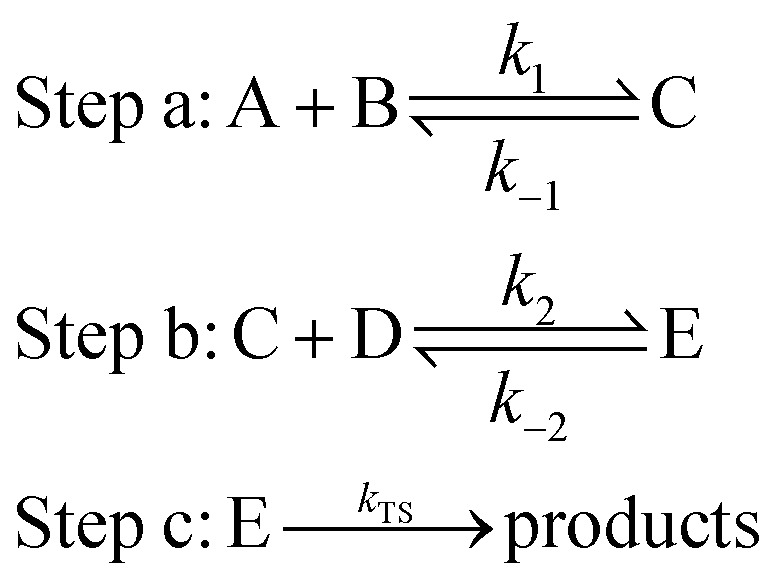
where A represents the reactant NH_3_ or OH, B represents (H_2_O)_*n*_ (*n* = 1–3), C represents a bimolecular complex, and *k*_1_ and *k*_−1_ are the forward and reverse rate constants, respectively, in step a. D represents another reactant, E represents the precursor complex, and *k*_2_ and *k*_−2_ are the corresponding forward and reverse rate constants, respectively, in step b. Step c is the reaction of the precursor complex with the transition state to form the products, and *k*_TS_ is the rate constant.

For this type of reaction, the total rate constant calculation for the involvement of water clusters can be written as:^22,37^

1 *k* = *K*_eq1_ × *K*_eq2_ × *k*_TS_

where *K*_eq1_ is the equilibrium constant between reactants and biomolecular complexes, *K*_eq2_ is the equilibrium constant between biomolecular complexes and trimolecular complexes, and *k*_TS_ is the reaction rate of the decisive speed step.^39,40^

The expression for the TST reaction rate constant^41^ for a reaction of A + B → C can be expressed as:2
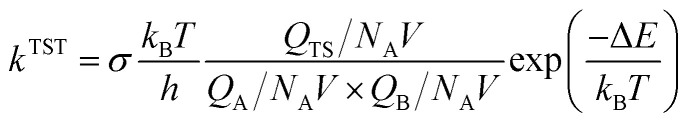
3
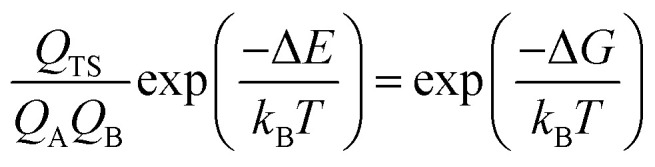
where *σ* is the system symmetry number, *k*_B_ is the Boltzmann constant, *T* is the absolute temperature and *h* is the Planck constant. *Q*_TS_, *Q*_A_ and *Q*_B_ represent the partition functions of transition state, reactant A and reactant B, respectively. *N*_A_ is Avogadro's constant, *V* is the volume, Δ*E* is the electron energy barrier, Δ*G* is the free energy barrier.

According to eqn (3), the TST equation can be changed to an equivalent thermodynamics form,4
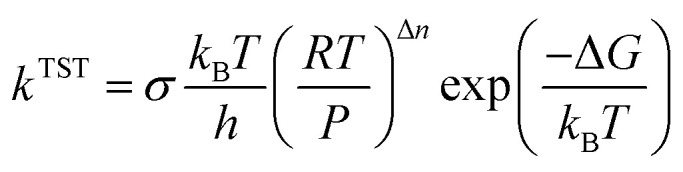
where *R* is the gas constant, *P* is the pressure, and for an *n*-molecule reaction, Δ*n* equals *n* minus 1. For reactions in the liquid phase, *G* denotes the solute free energy, which can be expressed as:^28^

5 *G*(liquid) = *G*_g_ + *G*_s_ + 1.89 kcal mol^−1^

where *G*(liquid) is the free energy in the solvent environment, *G*_g_ is the free energy in the gas-phase condition, and *G*_s_ is the solvation free energy under the implicit solvent model. The free energy correction of 1.89 kcal is derived from the change of free energy when 1 mole of solute molecules are dissolved from the gas phase to the liquid phase. Besides, the equilibrium constant (*K*_eq_) was given by eqn (6),6
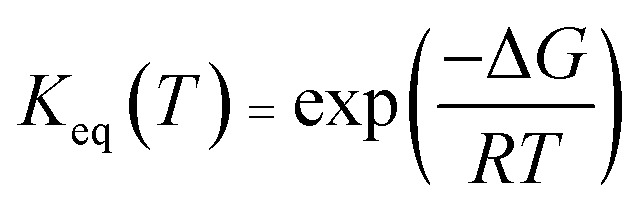


For the liquid phase reaction, the free energy is calculated using the self-consistent reaction field inside the Gaussian. In addition, the concentration of water molecules is relatively high relative to the concentration of A(OH/NH_3_) in the liquid phase environment, so the concentration of A·H_2_O is equal to the concentration of A. Therefore, the C[H_2_O]·*K*_eq1_ is close to 1 in the actual calculation. In the simplified case, we approximate the value of *K*_eq1_ to 1.

## 3. Results and discussion

The intermediate complexes in each reaction path are indicated by “IM” followed by a number, the transition states are denoted by “TS” followed by a number, and the intermediate final complexes are indicated by “IMF” followed by a number. Species with water monomers, water dimers and water trimers are indicated by the “WM”, “WD” and “WT” suffixes, respectively. The energy path diagram in this paper uses the reactant energy as the zero point.

### 3.1 Potential energy surfaces and rate constants for the reaction of NH_3_ + OH → NH_2_ + H_2_O

**Fig. 1:**
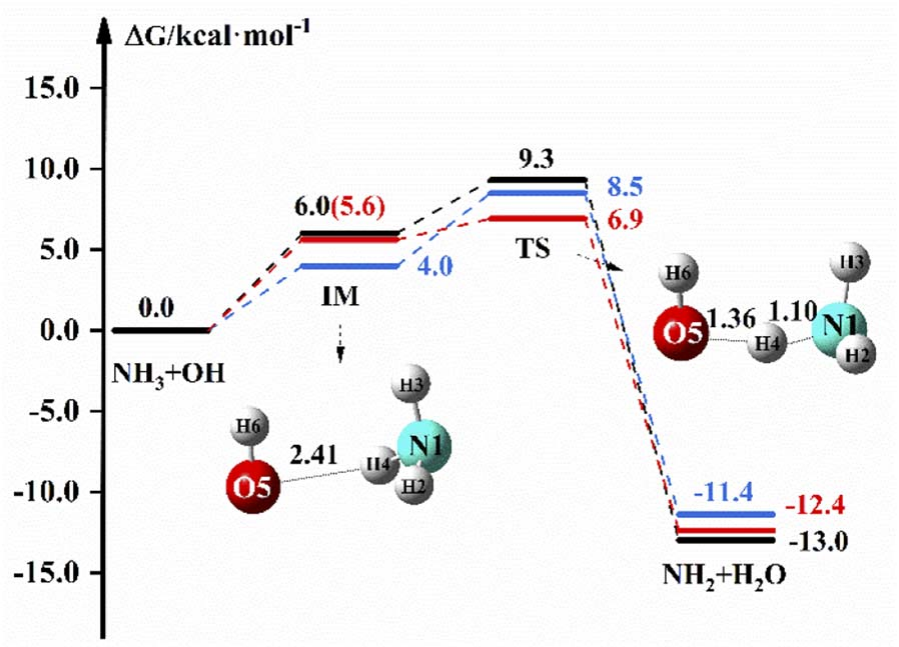
The free energy diagram for the NH_3_ + OH reaction at the M06-2X/6-311+G(2d,2p) level (blue line) and CCSD(T)-F12a/cc-pVDZ-F12//M06-2X/6-311+G(2d,2p) level (red line) in the gas phase and at the M06-2X/6-311+G(2d,2p) level under the implicit solvent model (black line).

Fig. 1 shows the reaction path of NH_3_ + OH → NH_2_ + H_2_O in the gas and liquid phase. In Fig. 1, the free energy of IM is 6.0 kcal mol^−1^ and the Δ*G* is 9.3 kcal mol^−1^ in the liquid phase. In the gas phase, the free energy of TS is 8.5 kcal mol^−1^ at the M06-2X/6-311+G(2d,2p) level, and the free energy of IM and TS are 5.6 and 6.9 kcal mol^−1^, respectively, at the CCSD(T)-F12a/cc-pVDZ-F12//M06-2X/6-311+G(2d,2p) level. The relevant data (electron energy, free energy) are listed on the ESI.†

Table 2 shows the rate constants both in the gas and liquid phases for the pure reaction. Δ*E*_TS_ is found to be 3.5 kcal mol^−1^ at the M06-2X/6-311+G(2d,2p) level in the gas phase, which is close to the previously reported value (3.3 kcal mol^−1^),^2^ and the rate constant is 2.07 × 10^−13^ cm^3^ molecule^−1^ s^−1^, which is in good agreement with the experimental value^20^ of 1.70 × 10^−13^ cm^3^ molecule^−1^ s^−1^. In the liquid phase, Δ*E*_TS_ is 3.7 kcal mol^−1^ at the M06-2X/6-311+G(2d,2p) level, and the rate constant calculated by using eqn (1) is 1.35 × 10^−13^ cm^3^ molecule^−1^ s^−1^, which is slightly less than the experimental value (5.84 × 10^−12^ cm^3^ molecule^−1^ s^−1^).^17^ In the gas phase, the rate constant is 3.47 × 10^−13^ cm^3^ molecule^−1^ s^−1^ at the CCSD(T)-F12a/cc-pVDZ-F12//M06-2X/6-311+G(2d,2p) level, which is not different from the theoretical^22^ and experimental^20^ results. The experimental results of the NH_3_ + OH reaction show that the rate constant of the liquid phase is 30 times larger than the rate constant of the gas phase, which indicates that the liquid phase conditions can accelerate the reaction of NH_3_ with OH. Our calculations show that the NH_3_ + OH reaction rate constants do not differ much between gas-phase and liquid-phase conditions.

**Table:** Rate constants (cm^3^ molecule^−1^ s^−1^) for the NH_3_ + OH → NH_2_ + H_2_O reaction at 298 K

	M06-2X		CCSD(T)		Exp
	Δ*E*_TS_	*k*	Δ*E*_TS_	*k*	*k*
Gas	3.5 (3.3)^a^	2.07 × 10^−13^	2.4 (3.3)^b^	3.47 × 10^−13^ (1.03 × 10^−13^)^b^	1.70 × 10^−13^^c^
Liquid	3.7	1.35 × 10^−13^	—		5.84 × 10^−12^^d^

a Ref. 2 M06-2X/aug-cc-pVQZ.

b Ref. 22 CCSD(T)-F12a/cc-pVDZ-F12.

c Ref. 20 Exp.

d Ref. 17 Exp; Δ*E*_TS_ (kcal mol^−1^) is the energy of the transition state relative to the intermediate complex. *k* is the total rate constant of the NH_3_ + OH reaction.

### 3.2 Potential energy surfaces and rate constants for the reaction of NH_3_ + OH + H_2_O → NH_2_ + H_2_O + H_2_O

**Fig. 2:**
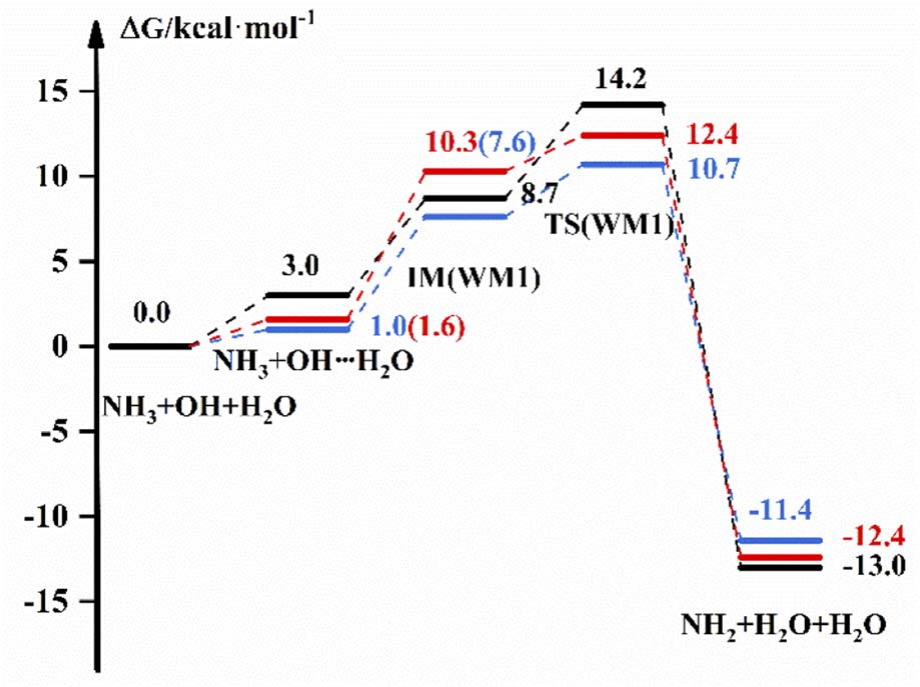
The free energy diagram for the NH_3_ + OH + H_2_O reaction at the M06-2X/6-311+G(2d,2p) level (blue line) and CCSD(T)-F12a/cc-pVDZ-F12//M06-2X/6-311+G(2d,2p) level (red line) in the gas phase and at the M06-2X/6-311+G(2d,2p) level under the implicit solvent model (black line).

The reaction scheme for the reaction of NH_3_ + OH with H_2_O is displayed in Fig. 2, which shows the free energies of the reactants, intermediate complexes, transition states and products by different methods. The intermediate complexes exist in different conformations, and we selected three conformations to study the effect of H_2_O on the pure reaction. Fig. 2 shows the reaction free energy diagram for one conformation; for the other two conformations, we have listed the reaction scheme in the ESI.†Fig. 2 shows that the free energy of the bimolecular complex and IM are 3.0 and 8.7 kcal mol^−1^, respectively, and the Δ*G* is 14.2 kcal mol^−1^ under the implicit solvent model. In the gas-phase environment, the free energy are 1.0 kcal mol^−1^ and 7.6 kcal mol^−1^, respectively, and Δ*G* is 10.7 kcal mol^−1^, which is 3.5 kcal mol^−1^ smaller than the result in liquid phase conditions. After we recalculate the energy of the reactants, intermediates, transition states and products in the gas-phase environment by using high-precision calculation methods, the *G* of IM is 10.3 kcal mol^−1^, and Δ*G* is 12.4 kcal mol^−1^. For WM3, NH_3_ binds more readily to H_2_O to form a complex, and the free energy of the bimolecular complex is 2.3 kcal mol^−1^. For other relevant data we see the ESI.† In addition, we give the transition state structures and precursor complex structures of the corresponding reaction path in Fig. S2 and S3.† Besides, we list the hydrogen bonding bond energies^42^ in the precursor complexes in Table S3.† Then, we calculate the rate constant for each path, and the results are listed in Table 3.

**Table:** Rate constants (cm^3^ molecule^−1^ s^−1^) for the NH_3_ + OH + H_2_O → NH_2_ + H_2_O + H_2_O reaction at 298 K^a^

		*K*_eq1_(WM)	*K*_eq2_(WM)	*k*_TS_(WM)	*k*(WM)
WM1	DFT(s)	1	6.06 × 10^−5^	3.77 × 10^−11^	2.28 × 10^−15^
	DFT(g)	1.88 × 10^−1^	1.47 × 10^−5^	2.08 × 10^−9^	5.75 × 10^−15^
	CC(g)	6.63 × 10^−2^	4.30 × 10^−7^	1.02 × 10^−8^	2.91 × 10^−16^
WM2	DFT(s)	1	2.35 × 10^−5^	9.27 × 10^−11^	2.18 × 10^−15^
	DFT(g)	1.78 × 10^−1^	1.34 × 10^−5^	2.44 × 10^−9^	5.83 × 10^−15^
	CC(g)	6.40 × 10^−2^	3.82 × 10^−7^	1.26 × 10^−8^	3.09 × 10^−16^
WM3	DFT(s)	1	3.18 × 10^−4^	1.05 × 10^−12^	3.35 × 10^−16^
	DFT(g)	1.83 × 10^−1^	4.15 × 10^−4^	2.20 × 10^−11^	1.67 × 10^−15^
	CC(g)	9.87 × 10^−2^	4.02 × 10^−5^	2.43 × 10^−11^	9.64 × 10^−17^

a *K*_eq1_(WM) is the equilibrium constant corresponding to step a, *K*_eq2_(WM) is the equilibrium constant corresponding to step b, and *k*_TS_(WM) is the rate constant corresponding to step c, *k*(WM) is the total rate constant for the NH_3_ + OH + H_2_O → NH_2_ + H_2_O + H_2_O reaction, s and g represent the calculation result in the liquid and gas phases.

In Table 3, we list the rate constants for the participation of H_2_O in the NH_3_ + OH reaction. And in this paper, the rate constants calculated using the free energy, and we list the free energy corresponding to each part of the NH_3_ + OH + (H_2_O)_*n*_ reaction in the ESI.† In real situations, the concentration of H_2_O is higher than the concentration of OH in the aqueous phase environment, which allows ‘step a’ to occur rapidly. From this information, we set the equilibrium constant of this part to 1 in the liquid phase, and *k*(WM) is the total rate constant obtained from the above. From Table 3, we observe that the addition of H_2_O under the implicit solvent model decreases the reaction rate constant by 2–3 orders of magnitude compared to the bare reaction rate constant of 1.35 × 10^−13^ cm^3^ molecule^−1^ s^−1^. The rate constants calculated by using the DFT method at the same level in the gas phase are 2 orders of magnitude lower than the bare reaction rate constant (2.07 × 10^−13^ cm^3^ molecule^−1^ s^−1^). Then, we further analyse the NH_3_ + OH + H_2_O reaction in the gas phase at the CCSD(T)-F12a/cc-pVDZ-F12//M06-2X/6-311+G(2d,2p) level, and the results show that the rate constant still decreases by 3–4 orders of magnitude, which is consistent with the theoretical results in the literature^22^ that the presence of H_2_O does not accelerate the NH_3_ + OH reaction. From Fig. S4,† the structures in the gas phase and liquid phase environments can be seen to be very different, which might result in differences in reaction rates across environments. The above results do not indicate that the liquid phase conditions promote the pure NH_3_ + OH reaction. In addition, the solvation effect becomes larger after the addition of H_2_O under the implicit solvent model. In the implicit solvent model, the solvation energy is composed mainly of the electrostatic interaction between solute and solvent, and the reactants OH, NH_3_ and water clusters have large polarity. In this way, the reduction in reactant energy caused by the solvation effect is greater than the reduction of reactant energy of the transition state, resulting in a reduction in the reaction rate. Of course, in the water environment, the translational and rotational free energy of reactants will increase, and the actual reaction energy barrier will decrease. Similarly, the translation and rotation of the solvent contribute to the reaction. On the other hand, the addition of water molecules to the NH_3_ + OH reaction results in an increase in the free energy of the reactants due to the low frequency and nonharmonic vibration^43–45^ modes of the water molecules. In addition to these factors, there may be other factors that can affect the rate. Considering these factors, the reaction rate will be higher than the current calculated value.

### 3.3 Potential energy surfaces and rate constants for the reaction of NH_3_ + OH + (H_2_O)_2_ → NH_2_ + H_2_O + (H_2_O)_2_

**Fig. 3:**
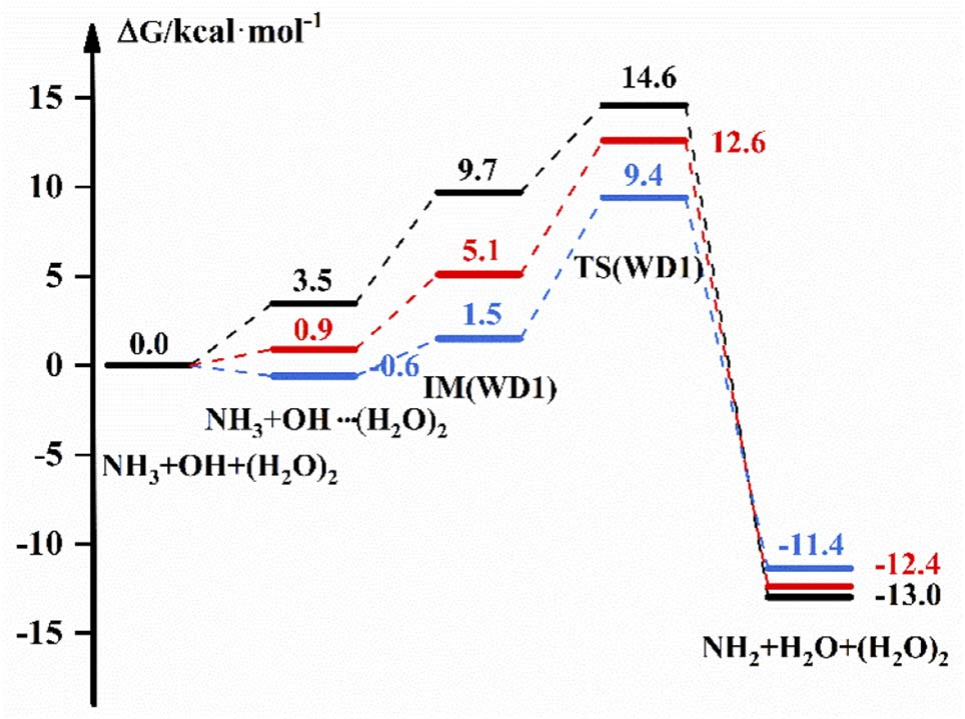
The free energy diagram for the NH_3_ + OH + (H_2_O)_2_ reaction at the M06-2X/6-311+G(2d,2p) level (blue line) and the CCSD(T)-F12a/cc-pVDZ-F12//M06-2X/6-311+G(2d,2p) level (red line) in the gas phase and at the M06-2X/6-311+G(2d,2p) level under the implicit solvent model (black line).

The free energy scheme for the NH_3_ + OH + (H_2_O)_2_ → NH_2_ + H_2_O + (H_2_O)_2_ reaction is displayed in Fig. 3. For WD1, Fig. 3 shows that the free energy of the complex formed by OH and (H_2_O)_2_ is 3.5 kcal mol^−1^, the *G* of IM is 9.7 kcal mol^−1^ and Δ*G* is 14.6 kcal mol^−1^ under the implicit solvent model. While in the gas phase, the free energy of the bimolecular complex is −0.6 kcal mol^−1^, the free energy of IM is 1.5 kcal mol^−1^ and Δ*G* is 9.4 kcal mol^−1^ with the DFT method. For WD2 and WD3 (Fig. S5†), (H_2_O)_2_ and NH_3_ are more likely to form complexes with free energies of 4.5 kcal mol^−1^ and 2.6 kcal mol^−1^ under the implicit solvent model, respectively. In addition, the corresponding energies for each part are also listed in Table S4,† and the transition state structures and precursor complex structures of the corresponding reaction path are given in Fig. S6 and S7.† Information such as electron energy and hydrogen bonding can also be obtained in the ESI.†

Table 4 shows the rate constant in the different environments after the addition of (H_2_O)_2_ to the NH_3_ + OH reaction. In the gas phase, the presence of (H_2_O)_2_ is decreased by 1 order of magnitude compared to the pure NH_3_ + OH reaction rate constant (2.07 × 10^−13^ cm^3^ molecule^−1^ s^−1^) by the DFT method, and the results corrected at the CCSD(T)-F12a/cc-pVDZ-F12 level also decreased by 2–4 orders of magnitude compared to the pure reaction rate constant. Under the implicit solvent model, the reaction rate constant is reduced by 2–3 orders of magnitude compared to the rate constant of 1.35 × 10^−13^ cm^3^ molecule^−1^ s^−1^. Similarly, the structural difference between the conformations in the gas-phase and liquid-phase environments is greater with the addition of water molecules, which is also responsible for the variability of the rate constants. After adding (H_2_O)_2_ to the NH_3_ + OH reaction, the probability of water clusters binding to radicals increases, and the multi-water effect is also more pronounced in the liquid phase than in the gas phase.

**Table:** Rate constants (cm^3^ molecule^−1^ s^−1^) for the NH_3_ + OH + (H_2_O)_2_ → NH_2_ + H_2_O + (H_2_O)_2_ reaction at 298 K^a^

		*K*_eq1_(WD)	*K*_eq2_(WD)	*k*_TS_(WD)	*k*(WD)
WD1	DFT(s)	1	2.80 × 10^−5^	9.63 × 10^−11^	2.70 × 10^−15^
	DFT(g)	2.70 × 10^0^	2.92 × 10^−2^	5.67 × 10^−13^	4.47 × 10^−14^
	CC(g)	2.24 × 10^−1^	7.63 × 10^−4^	1.25 × 10^−12^	2.14 × 10^−16^
WD2	DFT(s)	1	3.01 × 10^−3^	4.99 × 10^−13^	1.50 × 10^−15^
	DFT(g)	1.01 × 10^0^	1.30 × 10^0^	1.05 × 10^−14^	1.45 × 10^−14^
	CC(g)	1.91 × 10^−1^	2.12 × 10^−2^	2.23 × 10^−14^	9.07 × 10^−17^
WD3	DFT(s)	1	6.19 × 10^−5^	5.58 × 10^−12^	3.45 × 10^−16^
	DFT(g)	1.03 × 10^0^	9.37 × 10^−3^	6.56 × 10^−12^	6.36 × 10^−14^
	CC(g)	1.95 × 10^−1^	9.34 × 10^−4^	1.59 × 10^−11^	2.92 × 10^−15^

a *k*(WD) is the total rate constant for the NH_3_ + OH + (H_2_O)_2_ → NH_2_ + H_2_O + (H_2_O)_2_ reaction.

### 3.4 Potential energy surfaces and rate constants for the reaction of NH_3_ + OH + (H_2_O)_3_ → NH_2_ + H_2_O + (H_2_O)_3_

**Fig. 4:**
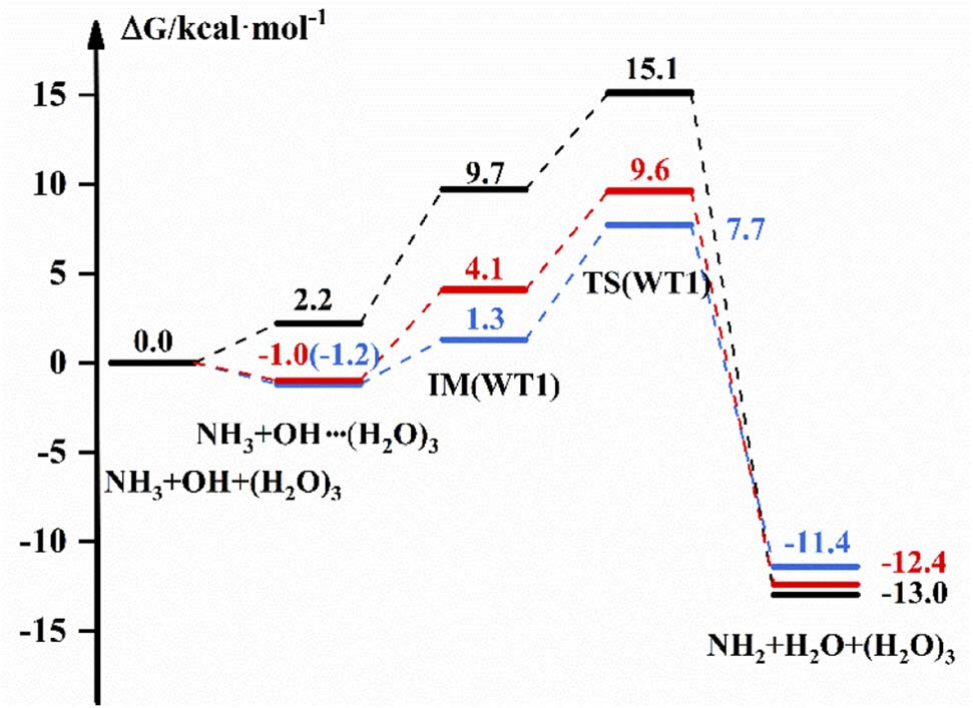
The free energy diagram for the NH_3_ + OH + (H_2_O)_3_ reaction at the M06-2X/6-311+G(2d,2p) level (blue line) and the CCSD(T)-F12a/cc-pVDZ-F12//M06-2X/6-311+G(2d,2p) level (red line) in the gas phase and at the M06-2X/6-311+G(2d,2p) level in the liquid phase (black line).

The free energy diagram for NH_3_ + OH + (H_2_O)_3_ are shown in Fig. 4 and S8.† From Fig. 4, the energy pathway of WT1 shows that the free energy of the bimolecular complex is 2.2 kcal mol^−1^, the free energy of IM is 9.7 and Δ*G* is 15.1 kcal mol^−1^ in the liquid phase. And in the gas phase, the Δ*G* of OH–(H_2_O)_3_ is −1.2 kcal mol^−1^, the free energy of IM is 1.3 kcal mol^−1^ and Δ*G* is 7.7 kcal mol^−1^ with the DFT method. In addition, the *G* of the bimolecular complex and IM are −1.0 and 4.1 kcal mol^−1^, respectively, and the Δ*G* is 9.6 kcal mol^−1^ at the CCSD(T)-F12a/cc-pVDZ-F12//M06-2X/6-311+G(2d,2p) level in the gas phase. And for WT3, the free energy of IM in the gas phase is 4.1 kcal mol^−1^ with the DFT method, which is 6.6 kcal mol^−1^ different from the results in the liquid phase, and the Δ*G* values are both smaller than those in the liquid phase. In addition, the ESI† provides structure diagrams, energies, hydrogen bonds, and other relevant data.

The reaction rate constants are listed in Table 5 to compare the effect of (H_2_O)_3_ on the NH_3_ + OH reaction rate constants. The rate constant is (1.03 × 10^−16^ to 1.57 × 10^−16^) cm^3^ molecule^−1^ s^−1^ after the addition of (H_2_O)_3_ in the liquid phase, which is 3 orders of magnitude lower than the pure NH_3_ + OH reaction rate constant. In the gas phase, the rate constant is reduced by 1–3 orders of magnitude compared to the original reaction at the CCSD(T)-F12a/cc-pVDZ-F12//M06-2X/6-311+G(2d,2p) level.

**Table:** Rate constants (cm^3^ molecule^−1^ s^−1^) for the NH_3_ + OH + (H_2_O)_3_ → NH_2_ + H_2_O + (H_2_O)_3_ reaction at 298 K^a^

		*K*_eq1_(WT)	*K*_eq2_(WT)	*k*_TS_(WT)	*k*(WT)
WT1	DFT(s)	1	3.26 × 10^−6^	4.14 × 10^−11^	1.35 × 10^−16^
	DFT(g)	7.44 × 10^0^	1.49 × 10^−2^	7.59 × 10^−12^	8.39 × 10^−13^
	CC(g)	4.96 × 10^0^	1.87 × 10^−4^	3.71 × 10^−11^	3.44 × 10^−14^
WT2	DFT(s)	1	4.15 × 10^−6^	2.49 × 10^−11^	1.03 × 10^−16^
	DFT(g)	1.18 × 10^−3^	8.07 × 10^−1^	4.91 × 10^−12^	4.69 × 10^−15^
	CC(g)	5.09 × 10^−4^	3.96 × 10^−3^	8.70 × 10^−11^	1.75 × 10^−16^
WT3	DFT(s)	1	2.49 × 10^−6^	6.32 × 10^−11^	1.57 × 10^−16^
	DFT(g)	1.14 × 10^−2^	4.23 × 10^−2^	7.46 × 10^−12^	3.60 × 10^−15^
	CC(g)	5.16 × 10^−3^	2.06 × 10^−4^	1.47 × 10^−10^	1.56 × 10^−16^

a *k*(WT) is the total rate constant for the NH_3_ + OH + (H_2_O)_3_ → NH_2_ + H_2_O + (H_2_O)_3_ reaction.

From Fig. S11,† the free energy of the bimolecular complexes is lower than the free energy of reactants, indicating that ‘step a’ can occur quickly in the actual reaction. The free energies of IM and TS are higher than the free energies of reactants; therefore, it is feasible to calculate the reaction rate constant by using eqn (1).

### 3.5 Potential energy surfaces and rate constants in the liquid phase for the reaction of NH_3_ + OH + (H_2_O)_13_ → NH_2_ + H_2_O + (H_2_O)_13_

**Fig. 5:**
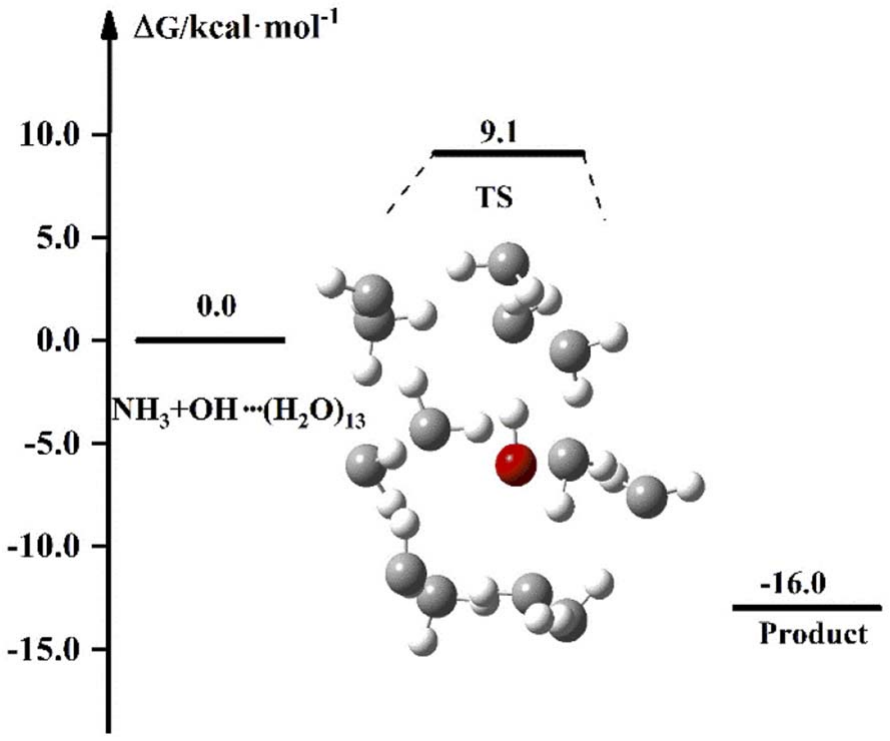
The free energy diagram for the NH_3_ + OH + (H_2_O)_13_ reaction at the M06-2X/6-311+G(2d,2p) level in the liquid phase.

The experimental results of the reaction of NH_3_ with OH show that the liquid phase conditions can accelerate the reaction. In the discussion of the previous sections, the results after the involvement of (H_2_O)_*n*_ (*n* = 1–3) in the NH_3_ + OH reaction showed that the liquid-phase environment did not promote the NH_3_ + OH reaction. The liquid phase environment is very complex, it is uncertain how many water clusters are involved in the reaction, in what form, and how many solvent molecules are required to achieve the solvent effect. In our calculations, since both OH and NH_3_ are polar molecules, the shielding effect of the first solvent shell on the reaction is significant. Therefore, we continued to increase the water clusters and did related tests for *n* = 5, *n* = 7, *n* = 8, *etc.* (the energy barriers and rate constants for the relevant reactions are shown in Table S8†). Then we found that when the water molecule was increased to 13, the dipole of a certain conformation changed and caused some effect on the rate. In the subsequent calculations, we did a study on the NH_3_ + OH reaction with the participation of (H_2_O)_13_, and Fig. 5 is the free energy diagram after the involvement of (H_2_O)_13_ in the NH_3_ + OH reaction. In Fig. 5, the free energy barrier of relative reactants for the reaction of NH_3_ and OH involving (H_2_O)_13_ is 9.1 kcal mol^−1^, which is less than the energy barrier (9.3 kcal mol^−1^) of the pure NH_3_ + OH reaction. This shows that the reaction of NH_3_ and OH can be promoted by the addition of (H_2_O)_13_ in the liquid phase. This is only one conformation, and there may be many more structures with the same effect as this conformation, as well as the conformation after the formation of the first solvent layer, and even the conformation of the higher solvent layers are certainly present. So we took (H_2_O)_13_ as an example to illustrate the shielding effect on the reaction rate.

**Fig. 6:**
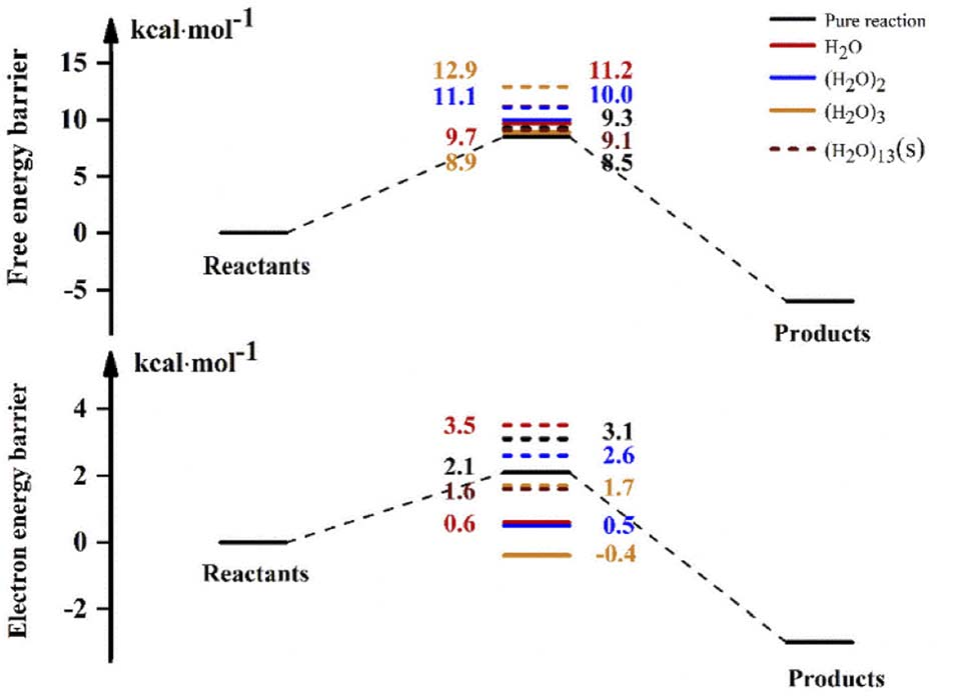
The electron and free energy barrier of relative reactants (NH_3_ + OH·(H_2_O)_*n*_) (kcal mol^−1^) in the gas and liquid phases. (H_2_O)_13_(s) denotes the energy barrier of adding (H_2_O)_13_ in the aqueous phase, and the solid and dashed lines represent the gas phase, respectively.

For the reaction in aqueous solution, the reactant is completely surrounded by water, and OH can be combined with aqueous clusters directly, that is, the equilibrium constant *K*_eq1_ is close to 1. In this way, the NH_3_ + OH reaction catalysed by water clusters in aqueous solution can be simplified to the bimolecular reaction of NH_3_ + OH·(H_2_O)_*n*_. We consider the NH_3_ and OH reactions involving (H_2_O)_13_ as ‘bimolecular reactions’, and for the convenience of comparison, we make the same approximation to the gas phase reaction. Then, the relative electron energy barrier, free energy barrier and reaction rate constants for the bimolecular reaction are listed in Table 6. At the same time, the corresponding reaction energy barrier diagrams are shown in Fig. 6. Here we did not calculate the total rate constant by eqn (1), but directly calculated the rate constant using the free energy barrier(Δ*G*′) of the transition state relative to the reactants (NH_3_ + OH·(H_2_O)_*n*_). *k*′ is the rate constant obtained using the above method. In Table 6, we can see that the rate constant after the addition of (H_2_O)_*n*_ (*n* = 1–3) in the gas phase is smaller than the original reaction rate constant, indicating that the water clusters in the gas phase cannot accelerate the NH_3_ + OH reaction. In the liquid phase, the pure reaction rate constant is 3.84 × 10^−14^ cm^3^ molecule^−1^ s^−1^, and this result is smaller than the experimental value (5.84 × 10^−12^ cm^3^ molecule^−1^ s^−1^) and the results (1.35 × 10^−13^ cm^3^ molecule^−1^ s^−1^) in Section 3.1, because the *k*′ calculated here is calculated directly using Δ*G*′ and does not consider the effect of precursor complex formation on the rate constant. In addition, the introduction of solvation electrostatic free energy will increase the energy barrier and reduce the reaction rate constant. In general, we can see from Table 6 that the rate constant of NH_3_ + OH·(H_2_O)_13_ in the liquid phase is 5.42 × 10^−14^ cm^3^ molecule^−1^ s^−1^, which is slightly larger than the rate constant (3.86 × 10^−14^ cm^3^ molecule^−1^ s^−1^) of the anhydrous reaction, this trend indicates that the liquid phase conditions promote the reaction of NH_3_ with OH.

**Table:** Electron energy barrier and free energy barrier (kcal mol^−1^)^a^

	Gas				Liquid			
	Δ*E*	Δ(*E +* ZPE)	Δ*G*′	*k*′	Δ*E*	Δ(*E +* ZPE)	Δ*G*′	*k*′
Pure reaction	2.7	2.1	8.5	1.49 × 10^−13^	3.5	3.1	9.3	3.86 × 10^−14^
(H_2_O)	0.1	0.6	9.7	2.10 × 10^−14^	4.2	3.5	11.2	1.56 × 10^−15^
(H_2_O)_2_	0.8	0.5	10.0	1.13 × 10^−14^	3.1	2.6	11.1	1.84 × 10^−15^
(H_2_O)_3_	−0.4	−0.4	8.9	7.71 × 10^−14^	1.3	1.7	12.9	8.85 × 10^−17^
(H_2_O)_13_	—	—	—	—	1.8	1.6	9.1	5.42 × 10^−14^

a Δ*E* (kcal mol^−1^) is the electron energy barrier relative to reactants (NH_3_ + OH·(H_2_O)_*n*_), Δ(*E +* ZPE) (kcal mol^−1^) is the electronic energy barrier including the zero-point energy, Δ*G*′ (kcal mol^−1^) is the free energy barrier relative to reactants (NH_3_ + OH·(H_2_O)_*n*_), *k*′ is the rate constant (cm^3^ molecule^−1^ s^−1^) obtained with Δ*G*′.

Actually, the participation of water clusters in the NH_3_ + OH reaction considers part of the explicit solvent effect, and there may be more promotion when other explicit solvent effects are considered. Since the conformation of the explicit phase, and there will be more phenomena in the actual reactions. In addition, considering that the translational and rotational degrees of freedom of solute in solvent will be limited to a certain extent, this factor leads to a slight decrease in the free energy of the transition state compared with the free energy of the ‘bimolecular’ reactant, which increases the reaction rate. However, this factor can be considered only with the help of an explicit solvent model, which requires many calculations. The reaction rate calculated by classical solvation theory and rate theory is still available. Also, we considered the participation of different water clusters in the NH_3_ + OH reaction, and in principle the participation of water in the reaction would have a problem with the standard state,^46–50^ there would be a solvation energy of water (4.3 kcal mol^−1^). However, the number of water molecules is constant throughout our reaction and the corrections caused by reactants, transition states and products can cancel each other, so we did not add an additional correction factor to the free energy.

### 3.6 The proton transfer of the reaction of NH_3_ + OH → NH_2_ + H_2_O in aqueous solution

**Fig. 7:**
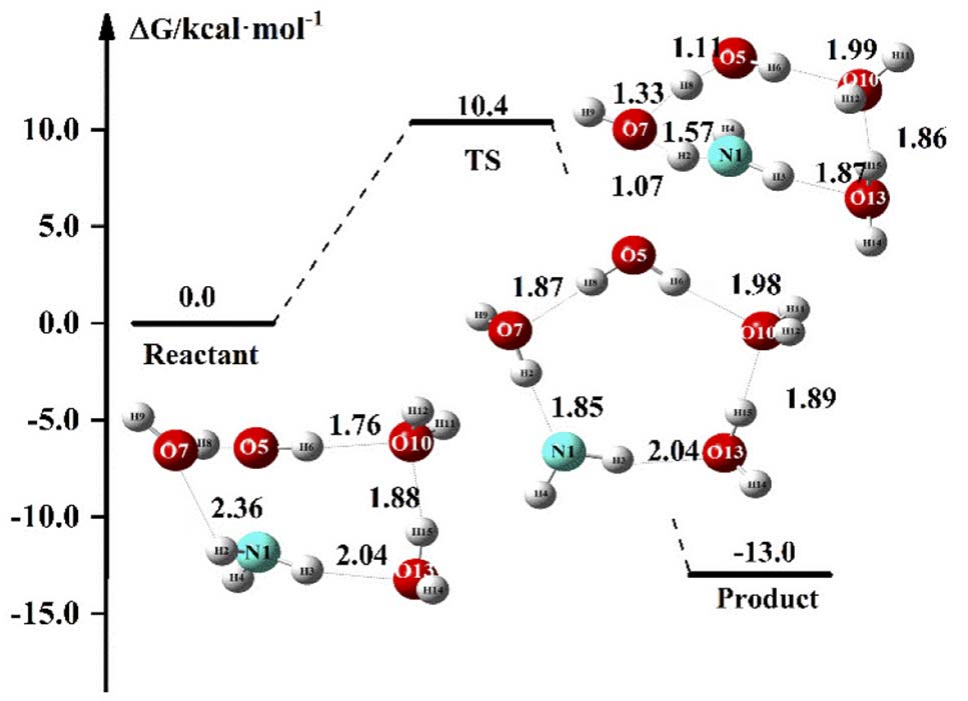
Proton transfer energy path in the NH_3_·OH·(H_2_O)_3_ reaction.

In the above discussion, NH_3_ can react directly with OH due to the strong oxidizing properties of OH. With the presence of numerous water molecules in aqueous solutions, OH may react with H_2_O. In this section, we discuss the proton transfer phenomenon of the NH_3_ + OH reaction in solution. Since the aqueous phase environment is very complex, we have discussed this issue using (H_2_O)_3_ as an example, and Fig. 7 shows the proton transfer energy path in the presence of (H_2_O)_3_ in the NH_3_ + OH reaction. Initially, NH_3_ is stably present in the NH_3_·OH·(H_2_O)_3_ complex. In the TS configuration, the strong oxidation of OH can clearly directly remove H from H_2_O, and at the same time, the newly formed OH will also remove H from NH_3_. The reaction of NH_3_ with OH is completed at the same time as the proton transfer.

In Table 7, we list the proton transfer rate constant and energy for each part of the NH_3_·OH·(H_2_O)_3_ reaction. The electron energy barrier and the free energy barrier for proton transfer in this reaction are 10.0 and 10.4 kcal mol^−1^, respectively, and the rate constant is 2.51 × 10^−16^ cm^3^ molecule^−1^ s^−1^. The proton transfer process usually has a relatively significant tunneling effect,^51^ which will further increase the reaction rate of NH_3_ + OH. The NH_3_·OH·(H_2_O)_3_ reaction pathway is another reaction mechanism between NH_3_ and OH in an aqueous environment. Compared to the liquid phase rate constant (1.35 × 10^−13^ cm^3^ molecule^−1^ s^−1^) in Section 3.1, the NH_3_ + OH reaction under this mechanism is 3 orders of magnitude slower. This reaction pathway is an additional reaction mechanism that is a purely facilitative effect on the NH_3_ + OH reaction in the liquid phase and provides a new mechanism, although the rate constants in the conformation are not high. The presence of this proton transfer-like reaction mechanism in the liquid-phase environment makes the reaction of NH_3_ with OH in aqueous much more probable. It shows that the aqueous phase environment promotes the NH_3_ + OH reaction, consistent with our experimental results. Since these two reaction mechanisms are different, we cannot directly correct the previous rate constants. In general, the aqueous phase environment promotes the reaction of NH_3_ with OH.

**Table:** Relative energy barrier (kcal mol^−1^) of each part in the reaction and the rate constant (cm^3^ molecule^−1^ s^−1^) of proton transfer at 298 K^a^

	TS	Product
Δ*E*	10.0	−12.7
Δ*G*	10.4	−13.5
*K*	2.51 × 10^−16^	

a Δ*E* is the electron energy relative to the reactant, Δ*G* is the free energy relative to the reactant, *k* is the proton transfer rate constant calculated using the free energy barrier.

In a water environment, the radical reaction of NH_3_ + OH, a reactant with large polarity, is very complex. Based on CCSD(T) and DFT methods, the gas-phase rate of this reaction is not different from the experimental value. Under the implicit solvent model, the liquid phase rate is lower than the experimental value. The reaction itself is approximately 30 times faster in the liquid phase than in the gas phase. When we consider the participation of (H_2_O)_*n*_ (*n* = 1–3) in the NH_3_ + OH reaction, the addition of (H_2_O)_*n*_ (*n* = 1–3) decreases the rate constant by 2–3 orders of magnitude and 0–2 orders of magnitude in the liquid and gas phases at the M06-2X/6-311+G(2d,2p) level and decreases the rate constant by 1–4 orders of magnitude at the CCSD(T)-F12a/cc-pVDZ-F12//M06-2X/6-311+G(2d,2p) level in the gas phase. The involvement of (H_2_O)_*n*_ (*n* = 1–3) in the reaction does not indicate that the liquid phase environment can promote the reaction of NH_3_ with OH. In the subsequent calculations, when *n* = 13, we can see that the rate constant is larger than the rate constant of the pure reaction, this trend indicates that the liquid phase conditions can accelerate the NH_3_ + OH reaction. In addition, the occurrence of proton transfer in solution also indicates that the liquid phase conditions can facilitate the NH_3_ + OH reaction. In summary, we used an implicit solvent model combined with a partially explicit solvent model to calculate the liquid-phase reaction rates. The liquid-phase reactions are very complex, so we approximated calculated the liquid-phase NH_3_ + OH reaction rate constants by considering the implicit solvent model, the addition of solvent molecules, and proton transfer. In the subsequent work, the calculation of the liquid-phase reaction can be further improved by using the explicit solvent model or other methods to obtain better results.

## 4. Conclusions

In order to consider the effect of aqueous solution on NH_3_ + OH reaction, we perform *ab initio* calculations for the water cluster-catalysed NH_3_ + OH reaction at the CCSD(T)-F12a/cc-pVDZ-F12//M06-2X/6-311+G(2d,2p) and M06-2X/6-311+G(2d,2p) levels for the gas phase and liquid phase, respectively. Firstly, we expect that the title reaction will show the characteristics of liquid phase reaction with the increase of the size of water clusters involved in the reaction. But the participation of (H_2_O)_*n*_ (*n* = 1–3) in the NH_3_ + OH reaction decreases the rate constants comparing with the pure reaction. This result is contrary to the experimental result that the liquid phase reaction rate is greater than the gas phase reaction rate. When we use the implicit solvent model to calculate the NH_3_ + OH reaction rate, the calculated results are still different from the experimental trend. Finally, we calculated the reaction rate with water clusters in the implicit solvent model. When the size of the water cluster surrounding OH radical is small, the theoretical reaction rate is still small. When the size of water cluster reaches *n* = 13, the calculated reaction rate begins to approach the experimental results. From the point of view of solvation model, both OH and NH_3_ have large dipole moments. The implicit solvent model is easy to overestimate the solvation free energy of reactants, resulting in significantly underestimating the reaction rate. When the water cluster surrounding the dipole increases, a more reasonable solvation energy correction will be obtained, and the calculated reaction rate is more reliable. In addition, our calculations also show that the proton transfer phenomenon present in the aqueous solution is also a factor that accelerates the reaction of NH_3_ with OH. The liquid phase reactions in real situations are very complex. Although this paper attempts to consider the role of various conformational water clusters and solvation electrostatic interactions, many conformations and other types of solvation effects are still not considered. For similar liquid phase reactions, more calculations need to be done in subsequent work, such as considering the first solvent layer or more solvent layers. For the implicit solvent model, the solvation radius of different atoms can be adjusted to make it suitable for the reaction involving free radicals, while the reasonable adjustment of the solvation radius requires more relevant calculations.

## Conflicts of interest

The authors declare no conflicts of interest.

## Supplementary Material

RA-012-D2RA04931G-s001
