# Supplementary material for: Effect of (H2O)n (n = 0–3, 13) on the NH3 + OH reaction in the gas and liquid phases
Source: RSC Adv. 2022 Sep 30;12(43):28010–9. doi: 10.1039/d2ra04931g (PMC9524257; doi:10.1039/d2ra04931g)
Supplement: RA-012-D2RA04931G-s001 [file RA-012-D2RA04931G-s001.pdf]

Electronic Supplementary Material (ESI) for RSC Advances.
This journal is © The Royal Society of Chemistry 2022

Supporting information
-for-

Effect Of $(H_2O)_n$(n=0-3,13) On The NH_3 + OH Reaction in the gas and liquid phases

Yujie Zhou[a], Qi Cao[b], Yu Yang[b], Dandan Ma[a], Quan Zhu[c,d] and Jianyi Ma[a,d]*

[a] Institute of Atomic and Molecular Physics, Sichuan University, Chengdu, 610065, China

[b] Reactor Operation and Application Research Sub-Institute, Nuclear Power Institute of China, Chengdu 610041, China

[c] School of Chemical Engineering, Sichuan University, Chengdu, 610065, PR China

[d] Engineering Research Center of Combustion and Cooling for Aerospace Power, Ministry of Education, Sichuan University, Chengdu, 610065, PR China

Table S1 Relative free energy (kcal·mol^{-1}) relative to reactants for NH_3+OH→NH_2+H_2O reaction.

	ΔG_{IM}	ΔG_{TS}
DFT(*s*)	6.0	9.3
DFT(*g*)	4.0	8.5
CCSD(T)(*g*)	5.6	6.9

ΔG_{IM} is the energy of the IM relative to the reactants and ΔG_{TS} is the energy of the TS relative to the reactants. *s* and *g* represent the calculation result in the liquid and gas phases.

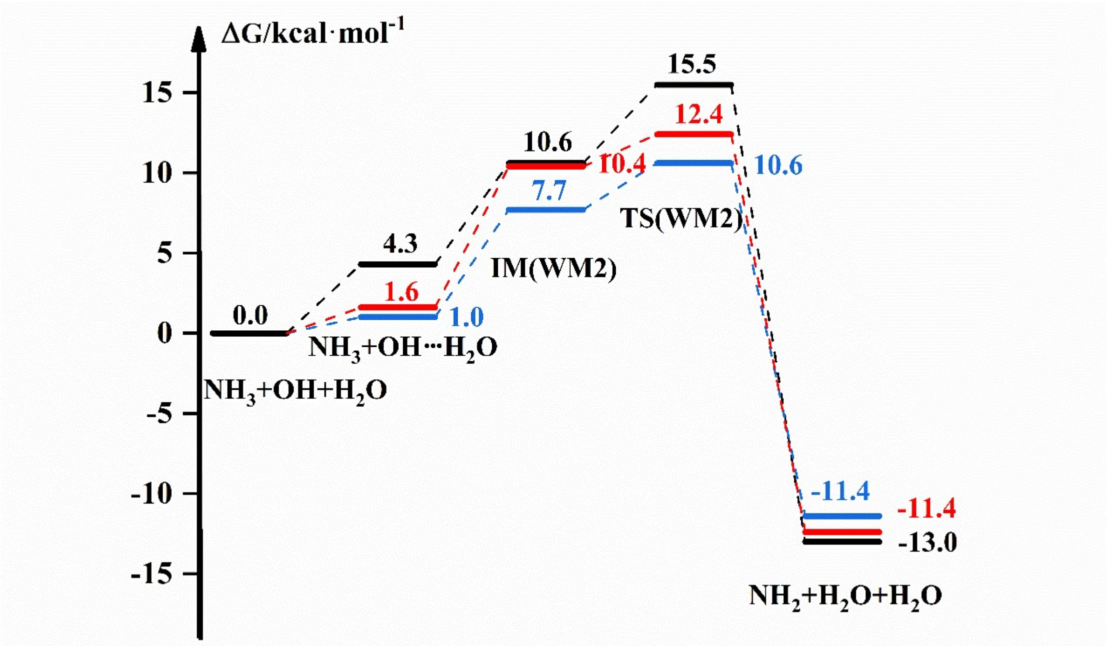

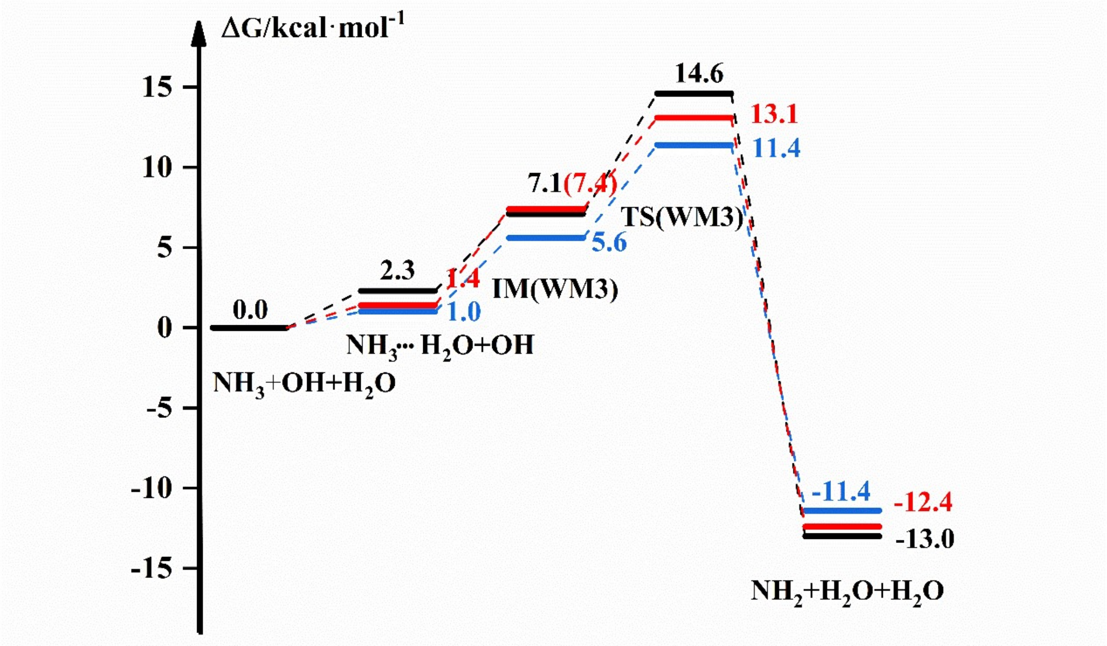

Fig. S1 The free energy diagram for the NH_3+OH+H_2O reaction at the M06-2X/6-311+G(2d,2p) level (blue line) and the CCSD(T)-F12a/cc-pVDZ-F12//M06-2X/6-311+G(2d,2p) level (red line) in the gas phase and at the M06-2X/6-311+G(2d,2p) level in the liquid phase (black line).

Table S2 Relative free energy (kcal·mol^{-1}) relative to reactants for $NH_3+OH+H_2O \rightarrow NH_2+H_2O+H_2O$ reaction.

		ΔG_1	ΔG_2	ΔG_3
	DFT(*s*)	3.0	8.7	14.2
WM1	DFT(*g*)	1.0	7.6	10.7
	CCSD(T)(*g*)	1.6	10.3	12.4
	DFT(*s*)	4.3	10.6	15.5
WM2	DFT(*g*)	1.0	7.7	10.6
	CCSD(T)(*g*)	1.6	10.4	12.4
	DFT(*s*)	2.3	7.1	14.6
WM3	DFT(*g*)	1.0	5.6	11.4
	CCSD(T)(*g*)	1.4	7.4	13.1

ΔG_1 is the energy of the bimolecular complex relative to the reactants and ΔG_2 is the energy of the IM relative to the reactants. ΔG_3 is the energy of TS relative to the reactants.

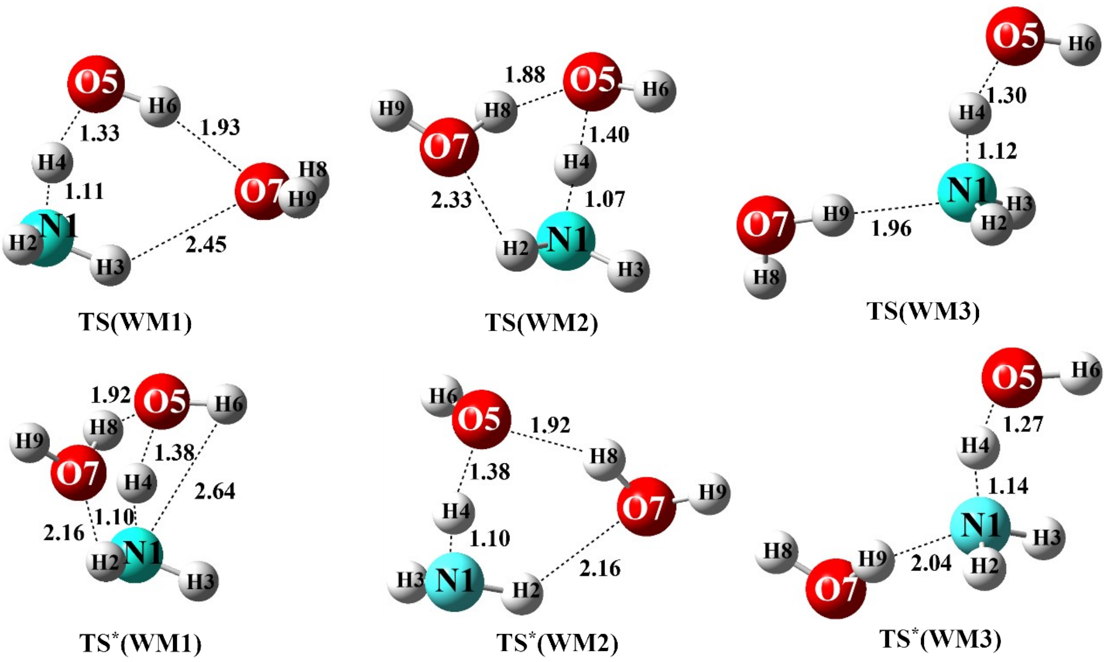

Fig. S2 The transition state structure of NH_3+OH+H_2O reaction at the M06-2X/6-311+G(2d,2p) level in the gas and liquid phases (bond length Å). The '*' indicates the transition state structure in the gas phase.

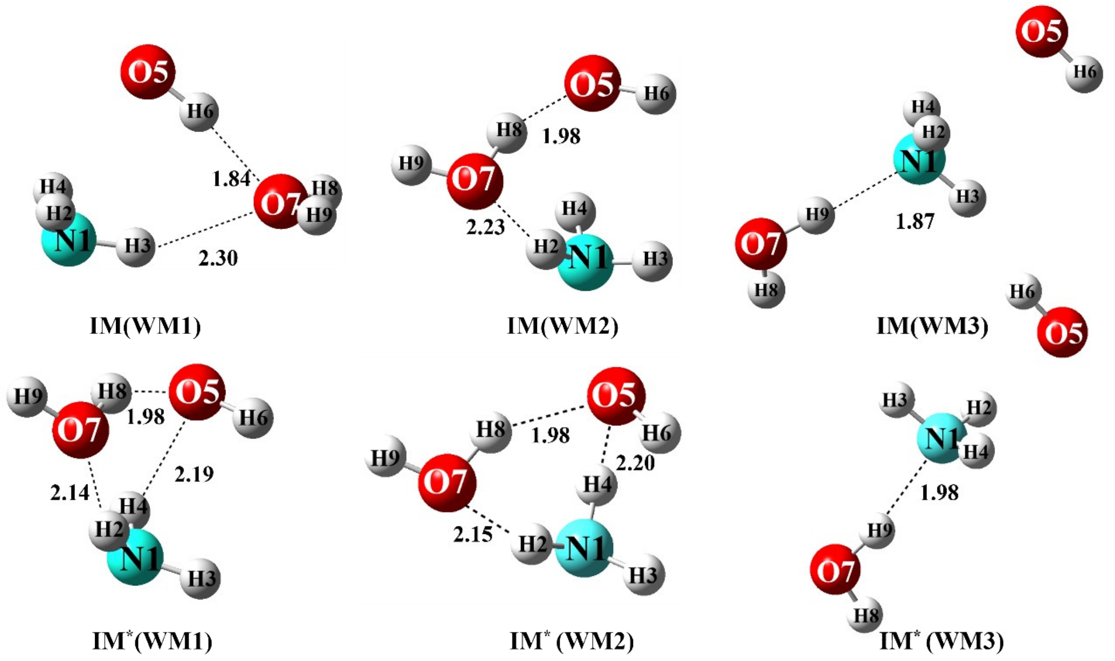

Fig. S3 The precursor complexes structure of NH_3+OH+H_2O reaction at the M06-2X/6-311+G(2d,2p) level in the gas and liquid phases (bond length Å). The '*' indicates the structure in the gas phase.

Table S3 Hydrogen bonding bond energy E_{HB} (kcal·mol^{-1}) in the precursor complex of the NH_3 + OH + H_2O reaction.

	liquid		gas	
		E_{HB}		E_{HB}
WM1			H2-O7	-2.45
	H6-O7	-4.37	H4-O5	-4.89
	H3-O7	-1.80	H8-O5	-3.49
WM2			H2-O7	-2.46
	H8-O5	-3.38	H4-O5	-4.86
	H2-O7	-2.07	H8-O5	-3.48
WM3	H9-N1	-4.73	H9-N1	-4.01

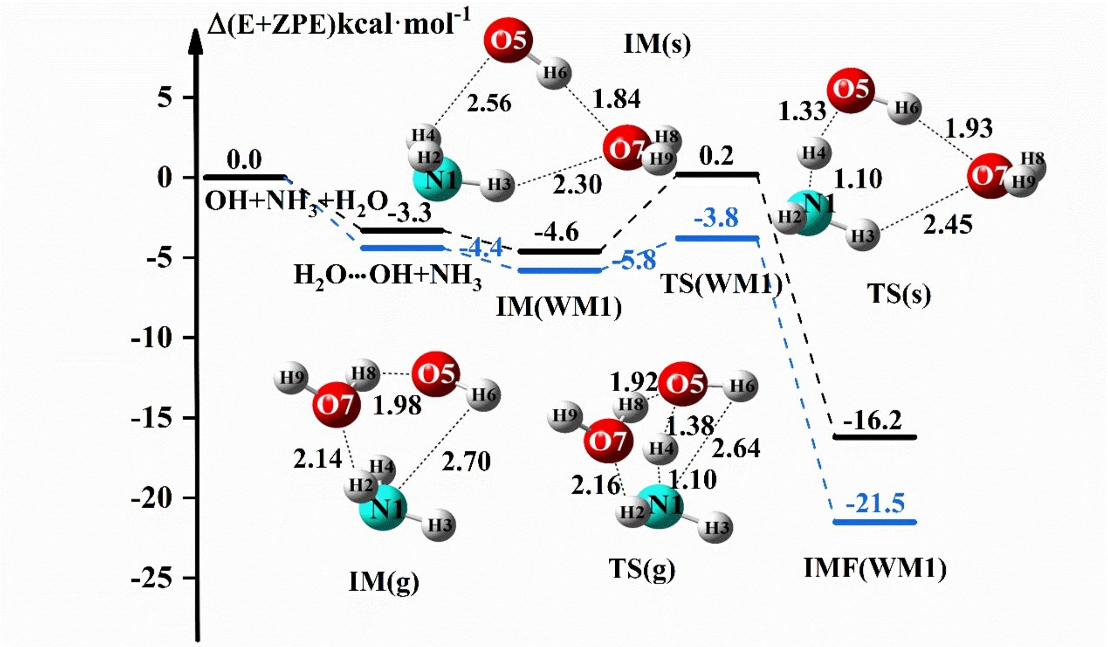

Fig. S4 Schematic energy diagram and optimized structures for WM1 at the M06-2X/6-311+G(2d,2p) level in the liquid (black line) and gas phases (blue line), *s* and *g* represent the calculation result in the liquid and gas phases (bond length Å).

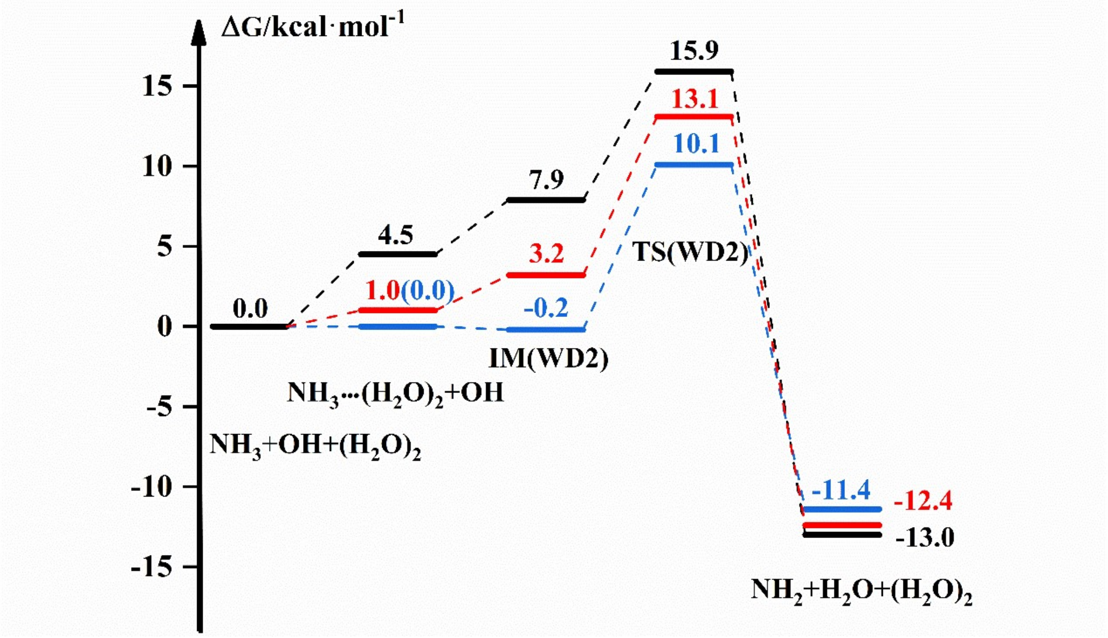

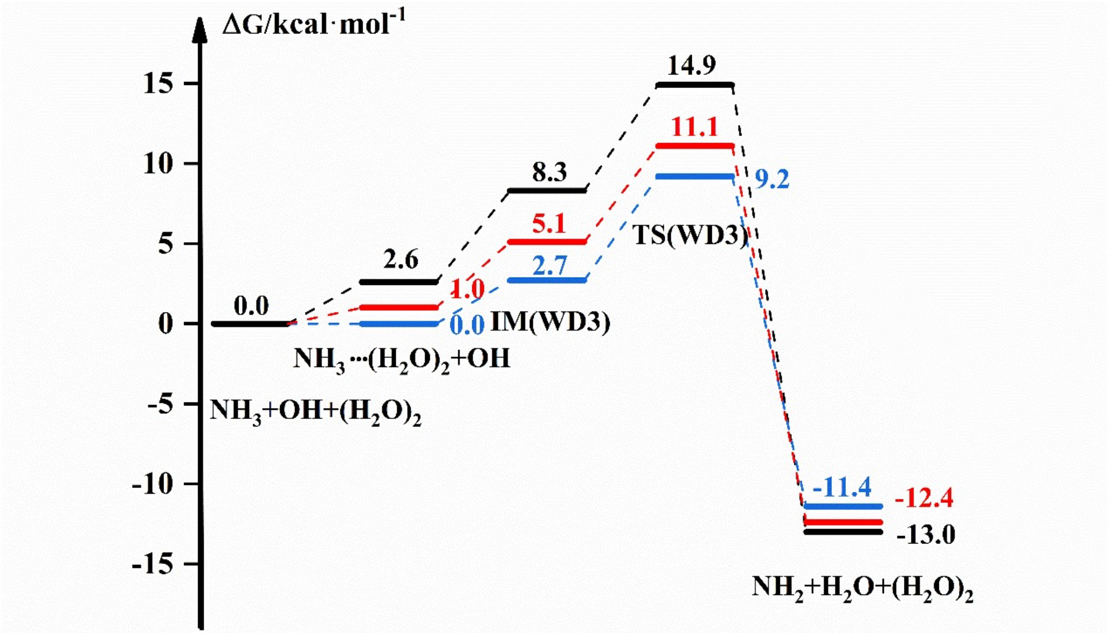

Fig. S5 The free energy diagram for the NH_3+OH+$(H_2O)_2$ reaction at the M06-2X/6-311+G(2d,2p) level (blue line) and the CCSD(T)-F12a/cc-pVDZ-F12//M06-2X/6-311+G(2d,2p) level (red line) in the gas phase and at the M06-2X/6-311+G(2d,2p) level in the liquid phase (black line).

Table S4 Relative free energy (kcal·mol^{-1}) relative to reactants for $NH_3+OH+(H_2O)_2 \rightarrow NH_2+H_2O+(H_2O)_2$ reaction.

		ΔG_1	ΔG_2	ΔG_3
	DFT(*s*)	3.5	9.7	14.6
WD1	DFT(*g*)	-0.6	1.5	9.4
	CCSD(T)(*g*)	0.9	5.1	12.6
	DFT(*s*)	4.5	7.9	15.9
WD2	DFT(*g*)	0.0	-0.2	10.1
	CCSD(T)(*g*)	1.0	3.2	13.1
	DFT(*s*)	2.6	8.3	14.9
WD3	DFT(*g*)	0.0	2.7	9.2
	CCSD(T)(*g*)	1.0	5.1	11.1

ΔG_1 is the energy of the bimolecular complex relative to the reactants and ΔG_2 is the energy of the IM relative to the reactants. ΔG_3 is the energy of TS relative to the reactants.

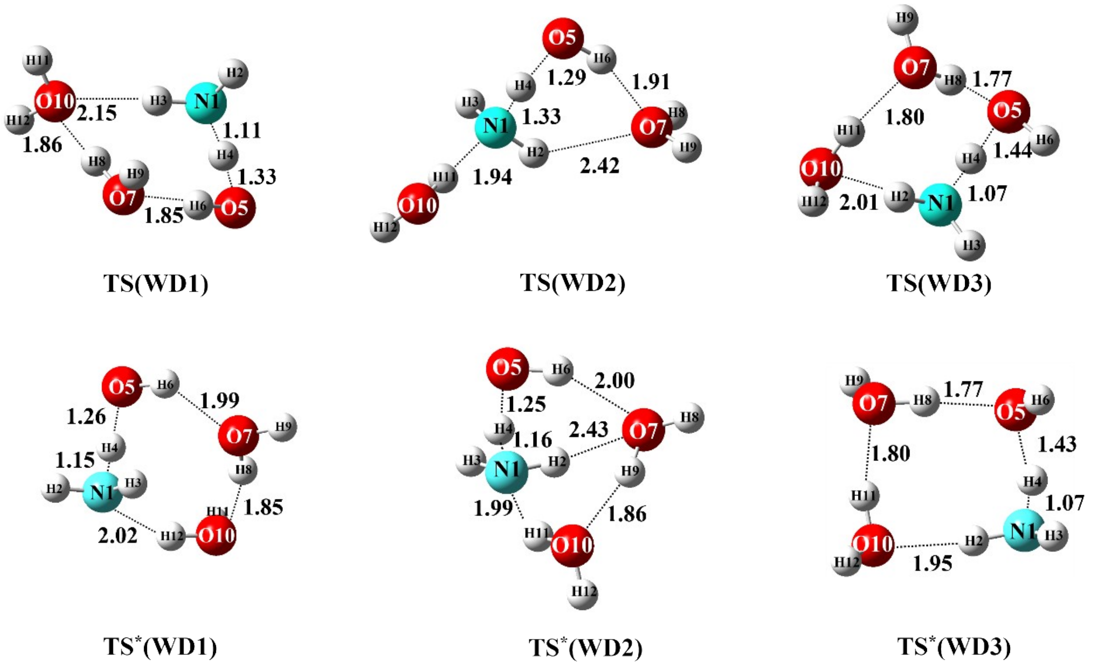

Fig. S6 The transition state structure of NH_3+OH+$(H_2O)_2$ reaction at the M06-2X/6-311+G(2d,2p) level in the gas and liquid phases (bond length Å). The '*' indicates the transition state structure in the gas phase.

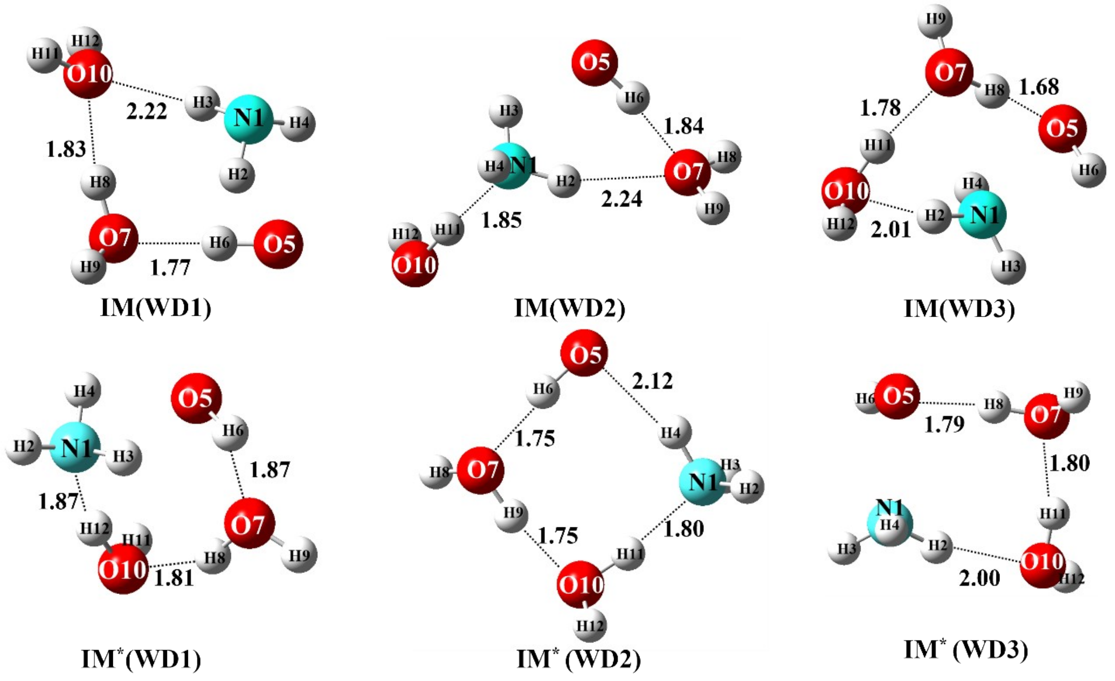

Fig. S7 The precursor complexes structure of NH_3+OH+$(H_2O)_2$ reaction at the M06-2X/6-311+G(2d,2p) level in the gas and liquid phases (bond length Å). The '*' indicates the structure in the gas phase.

Table S5 Hydrogen bonding bond energy(kcal·mol^{-1}) in the precursor complex of the NH_3 + OH + $(H_2O)_2$ reaction.

	liquid		gas	
		E_{HB}		E_{HB}
WD1	H3-O10	-2.13	H6-O7	-4.23
	H6-O7	-5.01	H8-O10	-5.03
	H8-O10	-4.46	H12-N1	-4.78
WD2			H4-O5	-2.56
	H2-O7	-2.08	H6-O7	-5.18
	H6-O7	-4.40	H9-O10	-5.26
	H11-N1	-4.90	H11-N1	-5.22
WD3	H2-O10	-3.37	H2-O10	-3.37
	H8-O5	-5.76	H8-O5	-4.64
	H11-O7	-5.09	H11-O7	-4.86

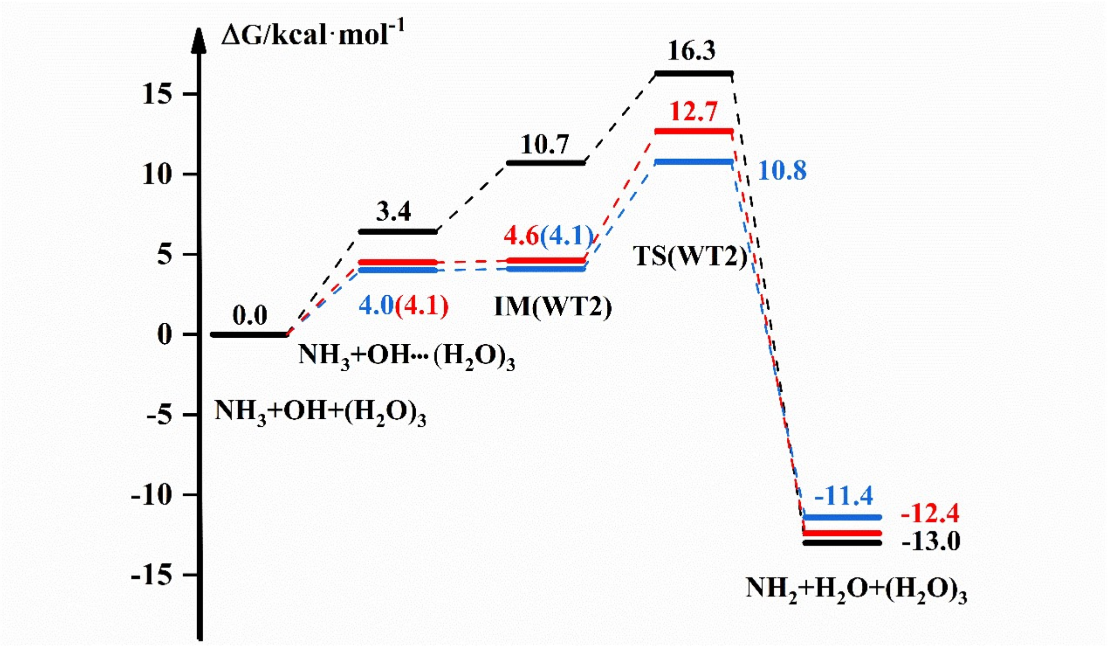

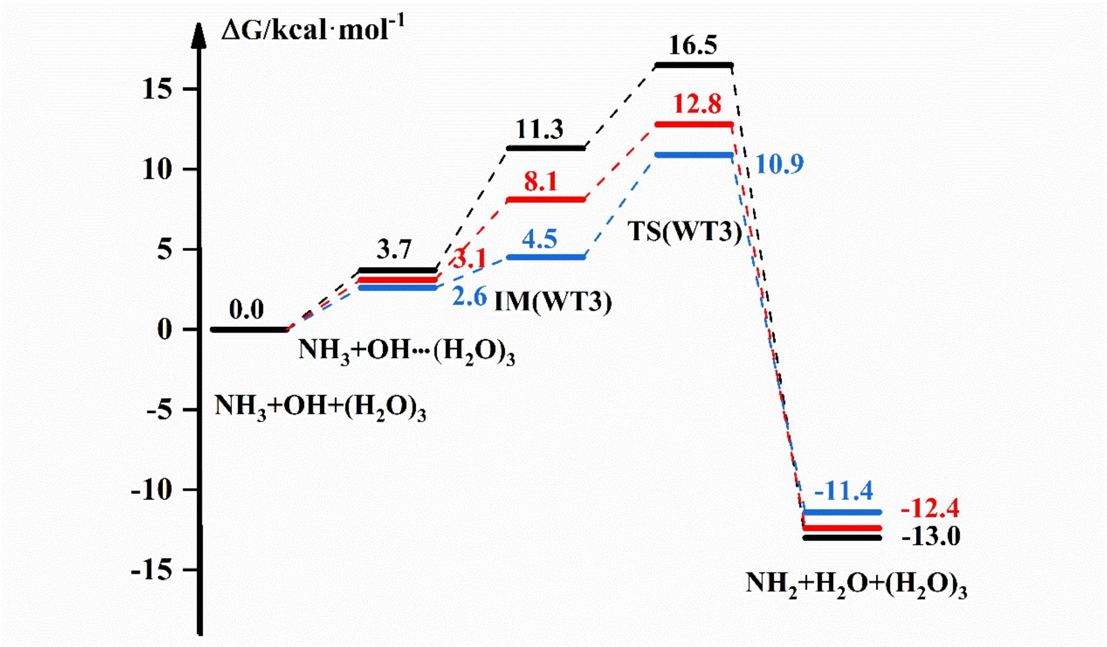

Fig. S8 The free energy diagram for the NH_3+OH+$(H_2O)_3$ reaction at the M06-2X/6-311+G(2d,2p) level (blue line) and the CCSD(T)-F12a/cc-pVDZ-F12//M06-2X/6-311+G(2d,2p) level (red line) in the gas phase and at the M06-2X/6-311+G(2d,2p) level in the liquid phase (black line).

Table S6 Relative free energy (kcal·mol^{-1}) relative to reactants for $NH_3+OH+(H_2O)_3 \rightarrow NH_2+H_2O+(H_2O)_3$ reaction.

		ΔG_1	ΔG_2	ΔG_3
	DFT(*s*)	2.2	9.7	15.1
WT1	DFT(*g*)	-1.2	1.3	7.7
	CCSD(T)(*g*)	-1.0	4.1	9.6
	DFT(*s*)	3.4	10.7	16.3
WT2	DFT(*g*)	4.0	4.1	10.8
	CCSD(T)(*g*)	4.5	4.1	12.7
	DFT(*s*)	3.7	11.3	16.5
WT3	DFT(*g*)	2.6	4.5	10.9
	CCSD(T)(*g*)	3.1	8.1	12.8

ΔG_1 is the energy of the bimolecular complex relative to the reactants and ΔG_2 is the energy of the IM relative to the reactants. ΔG_3 is the energy of TS relative to the reactants.

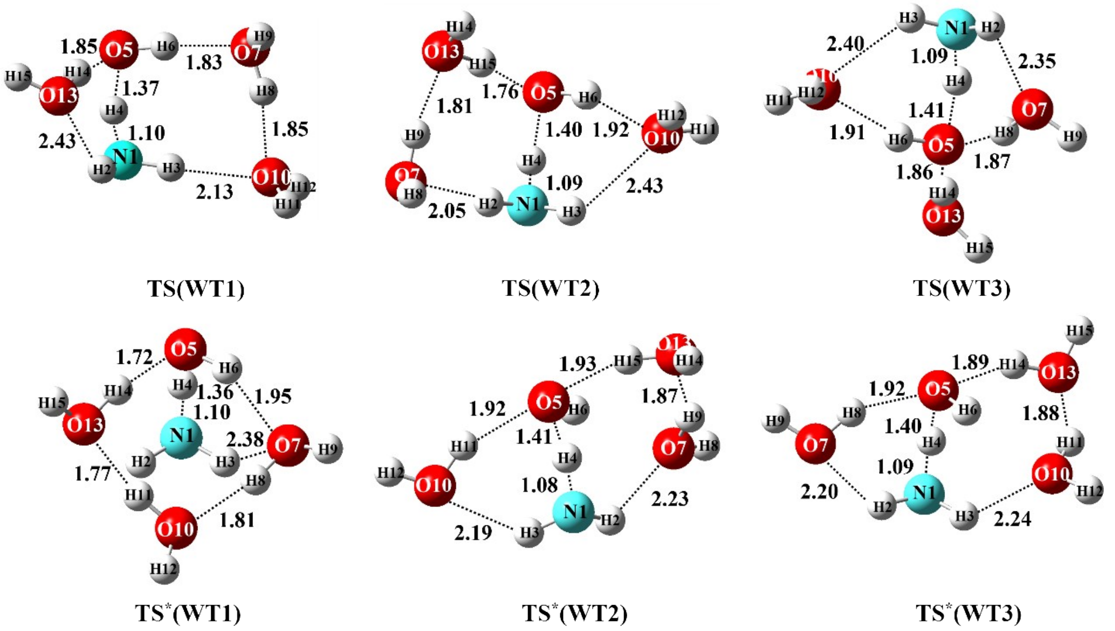

Fig. S9 The transition state structure of NH_3+OH+$(H_2O)_3$ reaction at the M06-2X/6-311+G(2d,2p) level in the gas and liquid phases (bond length Å). The '*' indicates the transition state structure in the gas phase.

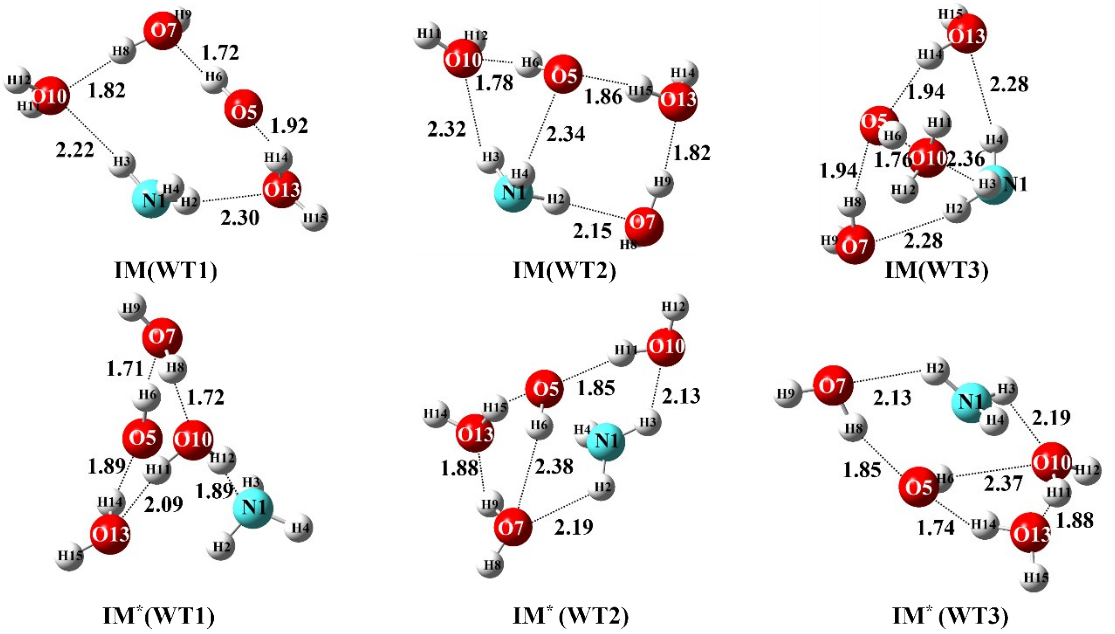

Fig. S10 The precursor complexes structure of NH_3+OH+$(H_2O)_3$ reaction at the M06-2X/6-311+G(2d,2p) level in the gas and liquid phases (bond length Å). The '*' indicates the structure in the gas phase.

Table S7 Hydrogen bonding bond energy(kcal·mol^{-1}) in the precursor complex of the NH_3 + OH + $(H_2O)_3$ reaction.

	liquid		gas	
		E_{HB}		E_{HB}
WT1	H2-O13	-1.77	H6-O7	-5.58
	H3-O10	-2.13	H8-O10	-5.72
	H6-O7	-5.42	H11-O13	-2.88
	H8-O10	-4.58	H12-N1	-4.51
	H14-O5	-3.76	H14-O5	-3.82
WT2	H2-O7	-2.54	H2-O7	-2.58
	H3-O10	-1.71	H3-O10	-2.72
	H4-O5	-2.37	H6-O7	-1.48
	H6-O10	-4.80	H9-O13	-4.18
	H9-O13	-4.65	H11-O5	-4.54
	H15-O5	-3.99	H15-O5	-5.57
WT3	H2-O7	-1.81	H2-O7	-2.73
	H3-O10	-1.52	H3-O10	-2.56
	H4-O13	-1.82	H6-O10	-1.50
	H6-O10	-5.04	H8-O5	-4.54
	H8-O5	-3.58	H11-O13	-4.18
	H14-O5	-3.57	H14-O5	-5.57

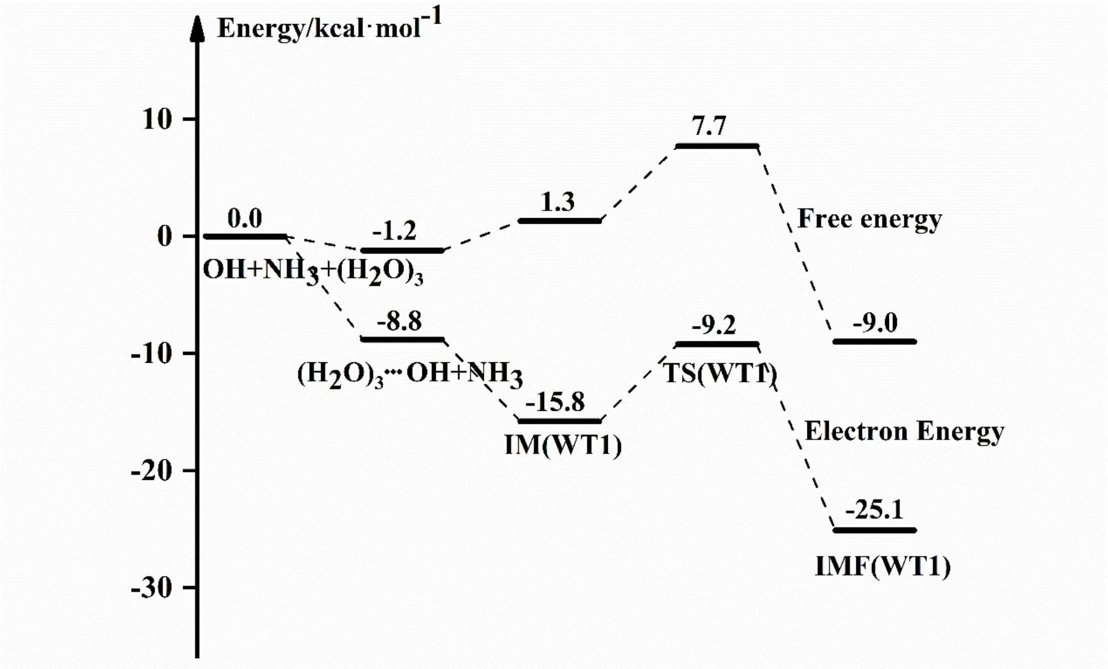

Fig. S11 The electronic Energy and the free energy for the NH_3+OH+$(H_2O)_3$ reaction at the M06-2X/6-311+G(2d,2p) level in the gas phase.

Table S8 Energy barriers(kcal·mol^{-1}) and rate constants(cm^3·molecules^{-1}·s^{-1}) for the reaction NH_3+OH+$(H_2O)_n$ in the liquid phase.

	ΔE	$\Delta(E+ZPE)$	ΔG	k'
$(H_2O)_5$	2.5	2.5	10.7	3.50E-15
$(H_2O)_7$	1.0	1.2	10.6	4.52E-15
$(H_2O)_8$	1.2	1.9	10.9	2.73E-15
$(H_2O)_{10}$	4.1	1.9	10.3	6.98E-15

ΔE is the electron energy barrier relative to reactants(NH_3+OH·$(H_2O)_n$), $\Delta(E+ZEP)$ is the electronic energy barrier including the zero-point energy, ΔG is the free energy barrier relative to reactants(NH_3+OH·$(H_2O)_n$), k' is the rate constant obtained with ΔG.

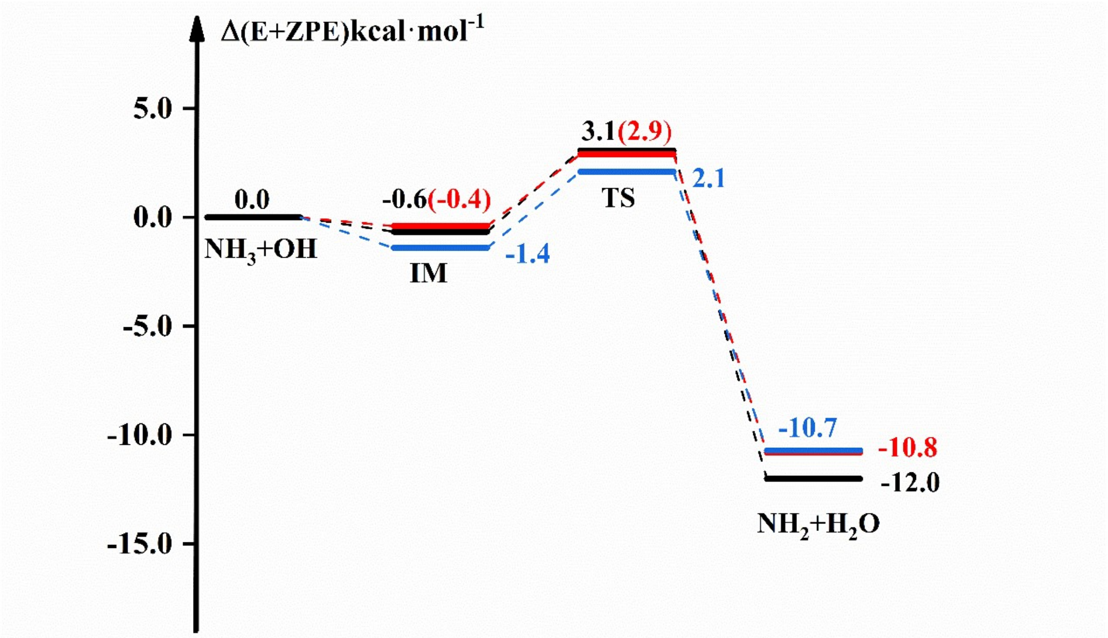

Fig. S12 Schematic energy diagram for the NH_3+OH reaction at the M06-2X/6-311+G(2d,2p) level(blue line) and the CCSD(T)-F12a/cc-pVDZ-F12// M06-2X/6-311+G(2d,2p) level (red line) in the gas phase, and at the M06-2X/6-311+G(2d,2p) level under the implicit solvent model (black line).

Table S9 Relative energy (kcal·mol^{-1}) relative to reactants for NH_3+OH→NH_2+H_2O reaction.

	ΔE_{IM}	ΔE_{TS}
DFT(*s*)	-0.6	3.1
DFT(*g*)	-1.4	2.1
CCSD(T)(*g*)	-0.4	2.9

ΔE_{IM} is the energy of the IM relative to the reactants and ΔE_{TS} is the energy of the TS relative to the reactants. *s* and *g* represent the calculation result in the liquid and gas phases.

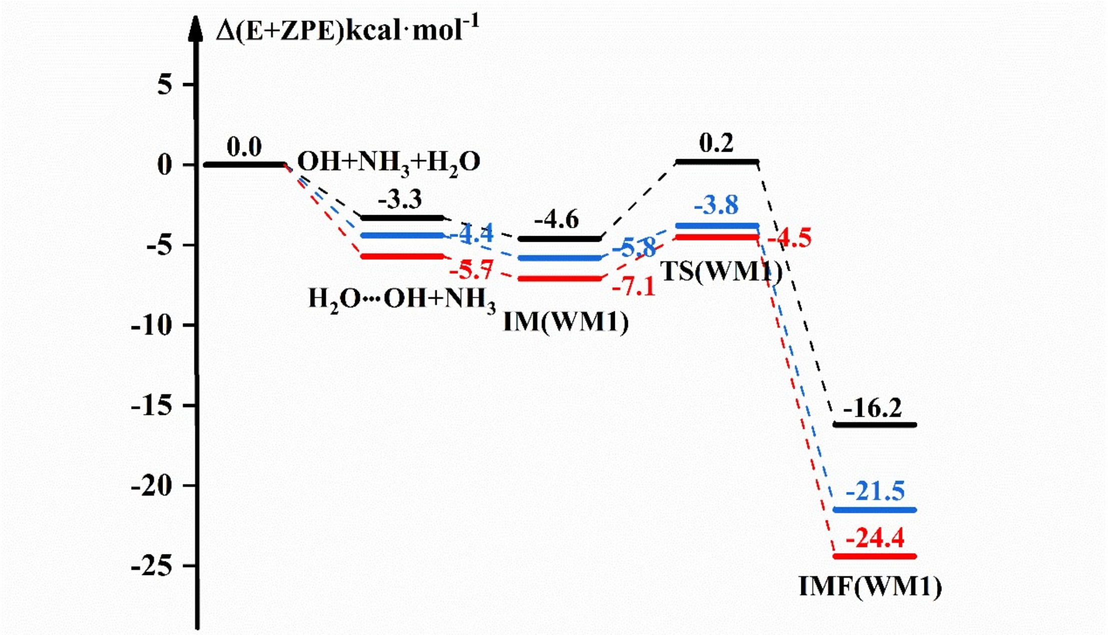

Δ(E+ZPE)kcal·mol-1
5
0
-5
-10
-15
-20
-25
0.0
OH+NH3+H2O
-3.3
-4.4
-5.7
H2O···OH+NH3
-4.6
-5.8
-7.1
IM(WM1)
0.2
-3.8
-4.5
TS(WM1)
-16.2
-21.5
-24.4
IMF(WM1)

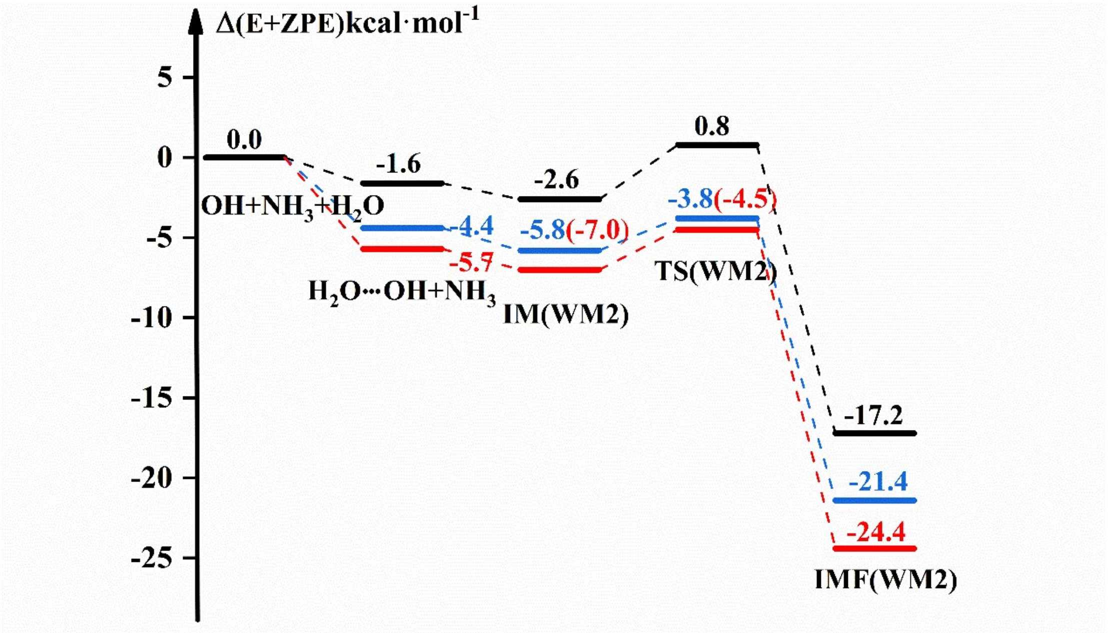

Δ(E+ZPE)kcal·mol-1
5
0
-5
-10
-15
-20
-25
0.0
OH+NH3+H2O
-1.6
-4.4
-5.7
H2O···OH+NH3
-2.6
-5.8(-7.0)
IM(WM2)
0.8
-3.8(-4.5)
TS(WM2)
-17.2
-21.4
-24.4
IMF(WM2)

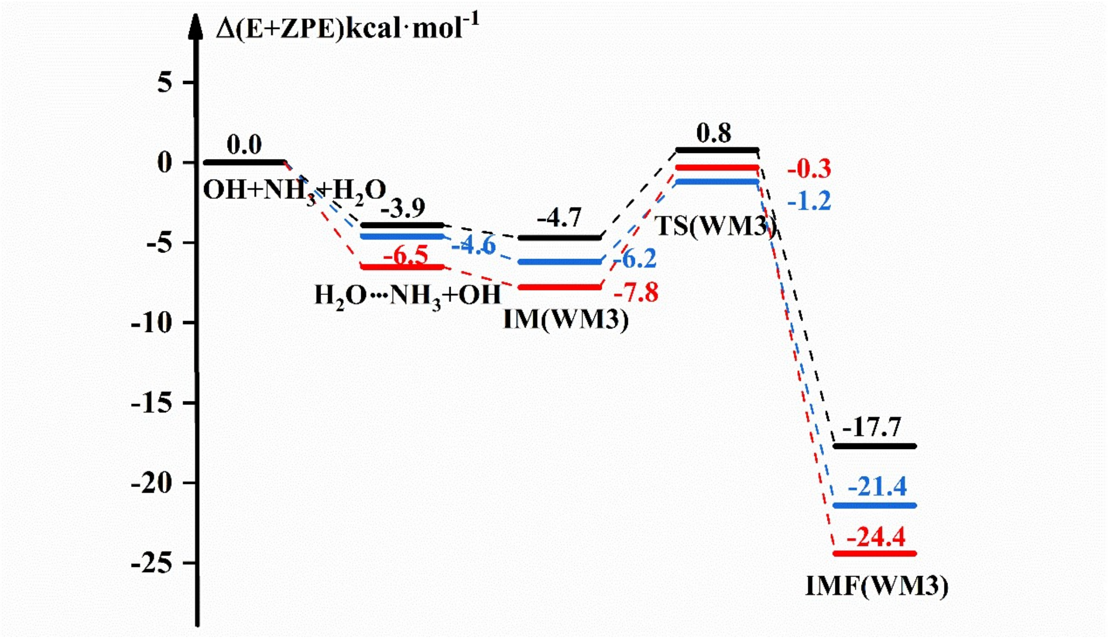

Fig. S13 Schematic energy diagram for the NH_3+OH+H_2O reaction at the M06-2X/6-311+G(2d,2p) level (blue line) and the CCSD(T)-F12a/cc-pVDZ-F12// M06-2X/6-311+G(2d,2p) level (red line) in the gas phase, and at the M06-2X/6-311+G(2d,2p) level under the implicit solvent model (black line).

Table S10 Relative electron energy (kcal·mol^{-1}) relative to reactants for NH_3+OH+H_2O→NH_2+H_2O+H_2O reaction.

		ΔE_1	ΔE_2	ΔE_3
	DFT(*s*)	-3.3	-4.6	0.2
WM1	DFT(*g*)	-4.4	-5.8	-3.8
	CCSD(T)(*g*)	-5.7	-7.1	-4.5
	DFT(*s*)	-1.6	-2.6	0.8
WM2	DFT(*g*)	-4.4	-5.8	-3.8
	CCSD(T)(*g*)	-5.7	-7.0	-4.5
	DFT(*s*)	-3.9	-4.7	0.8
WM3	DFT(*g*)	-4.6	-6.2	-1.2
	CCSD(T)(*g*)	-6.5	-7.8	-0.3

ΔE_1 is the energy of the bimolecular complex relative to the reactants and ΔE_2 is the energy of the IM relative to the reactants. ΔE_3 is the energy of TS relative to the reactants.

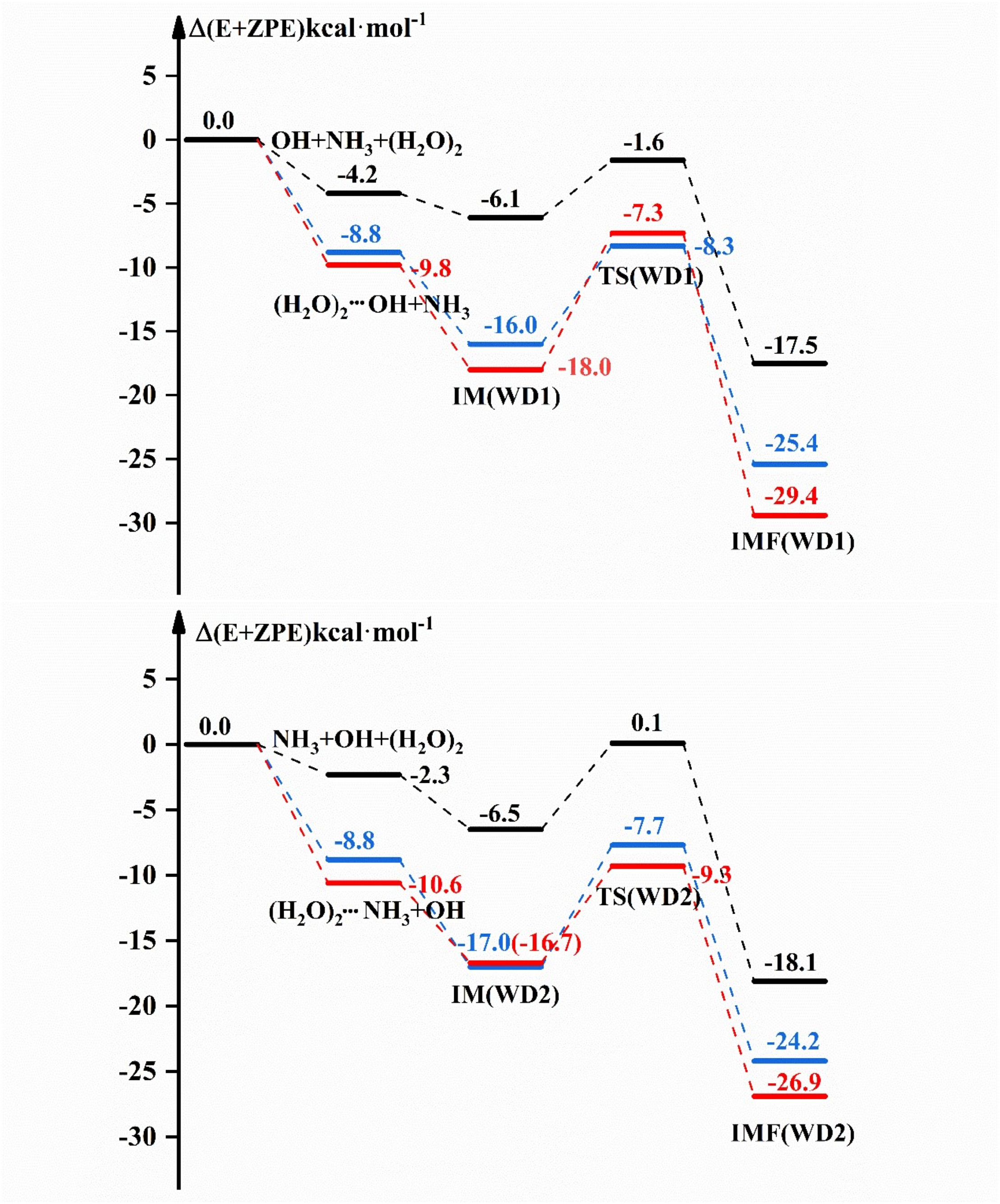

Δ(E+ZPE)kcal·mol-1
5
0
-5
-10
-15
-20
-25
-30
0.0
OH+NH3+(H2O)2
-4.2
-6.1
-1.6
-7.3
-8.8
-9.8
-8.3
TS(WD1)
(H2O)2···OH+NH3
-16.0
-18.0
-17.5
IM(WD1)
-25.4
-29.4
IMF(WD1)
Δ(E+ZPE)kcal·mol-1
5
0
-5
-10
-15
-20
-25
-30
0.0
NH3+OH+(H2O)2
0.1
-2.3
-6.5
-7.7
-8.8
-10.6
-9.3
TS(WD2)
(H2O)2··· NH3+OH
-17.0(-16.7)
-18.1
IM(WD2)
-24.2
-26.9
IMF(WD2)

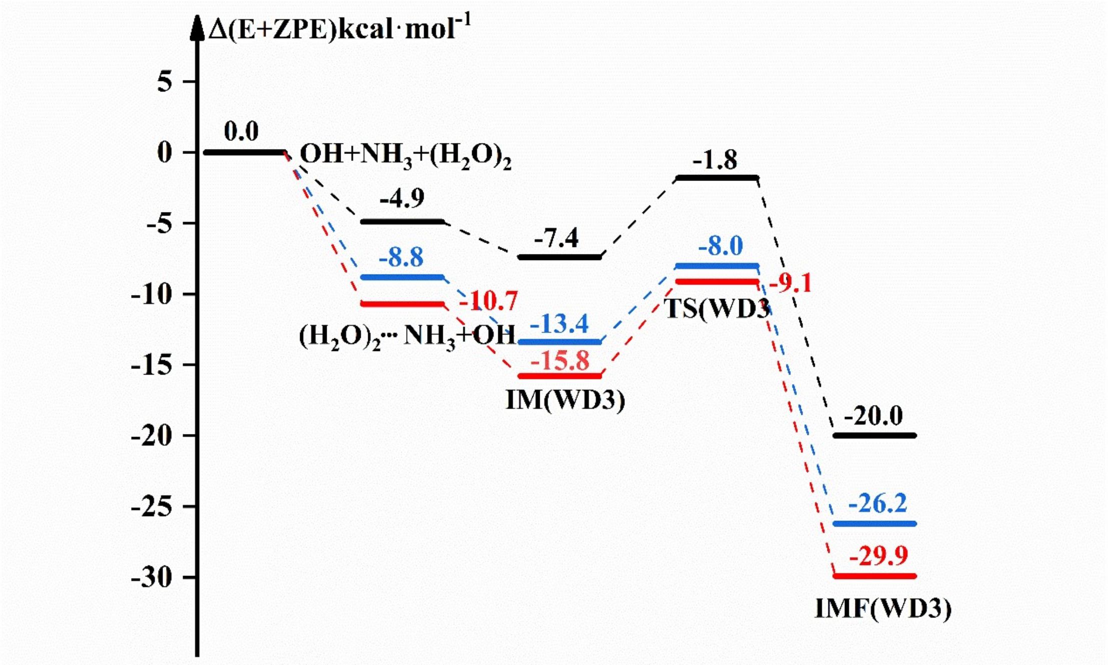

Fig. S14 Schematic energy diagram for the NH_3+OH+$(H_2O)_2$ reaction at the M06-2X/6-311+G(2d,2p) level (blue line) and the CCSD(T)-F12a/cc-pVDZ-F12// M06-2X/6-311+G(2d,2p) level (red line) in the gas phase, and at the M06-2X/6-311+G(2d,2p) level under the implicit solvent model (black line).

Table S11 Relative energy (kcal·mol^{-1}) relative to reactants for NH_3+OH+$(H_2O)_2$→NH_2+H_2O+$(H_2O)_2$ reaction.

		ΔE_1	ΔE_2	ΔE_3
	DFT(*s*)	-4.2	-6.1	-1.6
WD1	DFT(*g*)	-8.8	-16.0	-8.3
	CCSD(T)(*g*)	-9.8	-18.0	-7.3
	DFT(*s*)	-2.3	-6.5	0.1
WD2	DFT(*g*)	-8.8	-17.0	-7.7
	CCSD(T)(*g*)	-10.6	-16.7	-9.3
	DFT(*s*)	-4.9	-7.4	-1.8
WD3	DFT(*g*)	-8.8	-13.4	-8.0
	CCSD(T)(*g*)	-10.7	-15.8	-9.1

ΔE_1 is the energy of the bimolecular complex relative to the reactants and ΔE_2 is the energy of the IM relative to the reactants. ΔE_3 is the energy of the TS relative to the reactants.

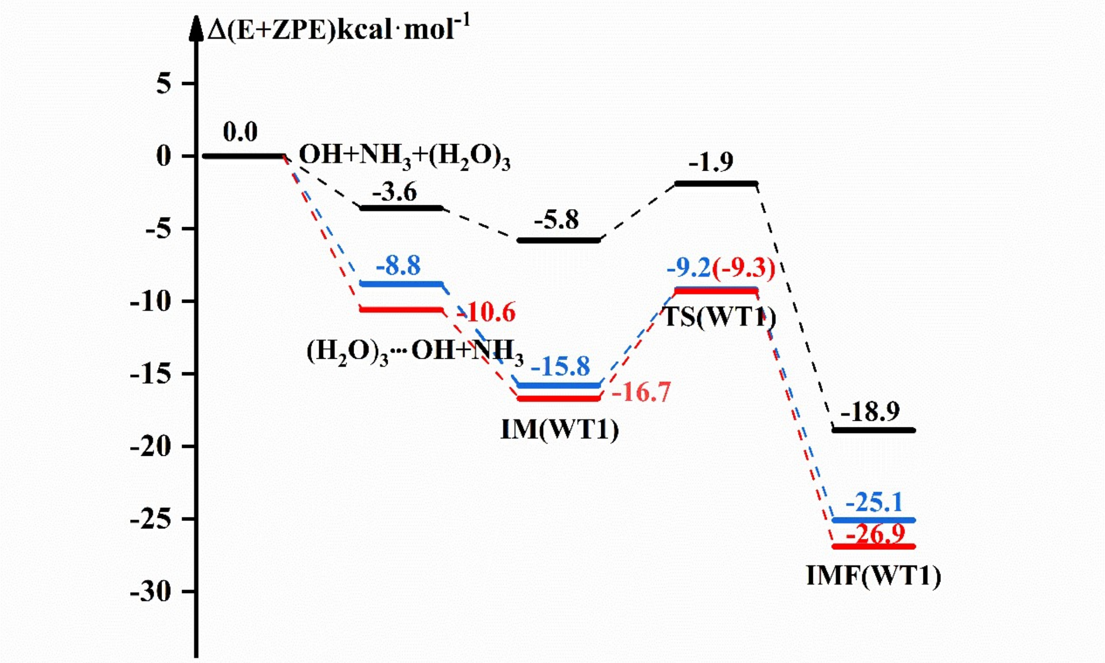

Δ(E+ZPE)kcal·mol-1
5
0
-5
-10
-15
-20
-25
-30
0.0
OH+NH3+(H2O)3
-3.6
-5.8
-1.9
-8.8
-10.6
-9.2(-9.3)
TS(WT1)
(H2O)3···OH+NH3
-15.8
-16.7
IM(WT1)
-18.9
-25.1
-26.9
IMF(WT1)

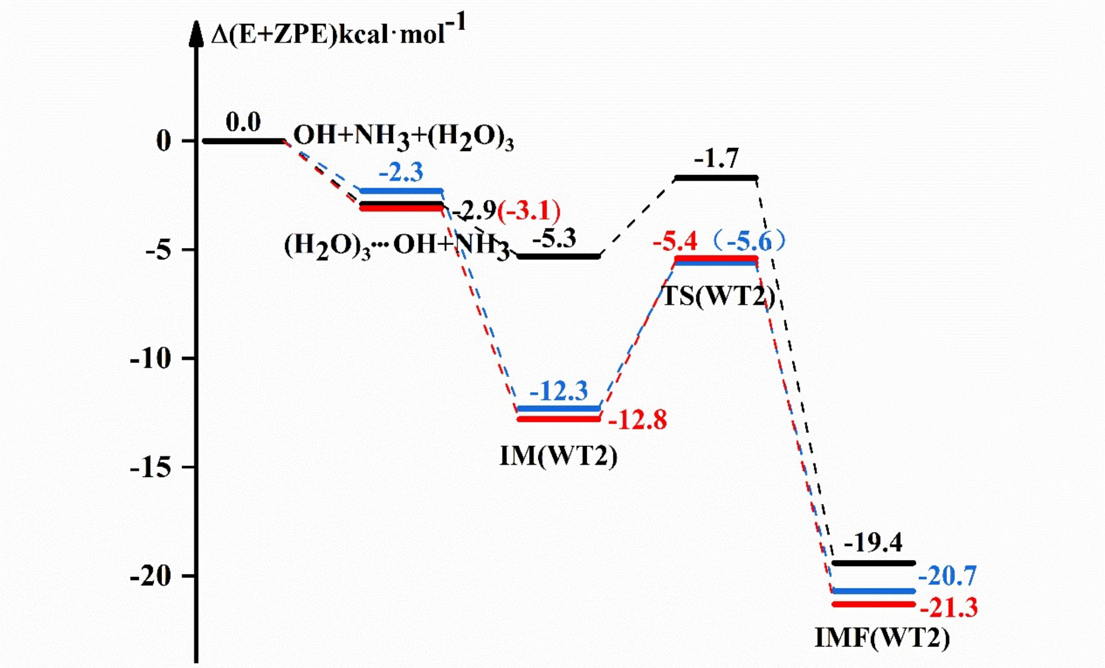

Δ(E+ZPE)kcal·mol-1
0
-5
-10
-15
-20
0.0
OH+NH3+(H2O)3
-2.3
-2.9(-3.1)
(H2O)3···OH+NH3
-5.3
-1.7
-5.4 (-5.6)
TS(WT2)
-12.3
-12.8
IM(WT2)
-19.4
-20.7
-21.3
IMF(WT2)

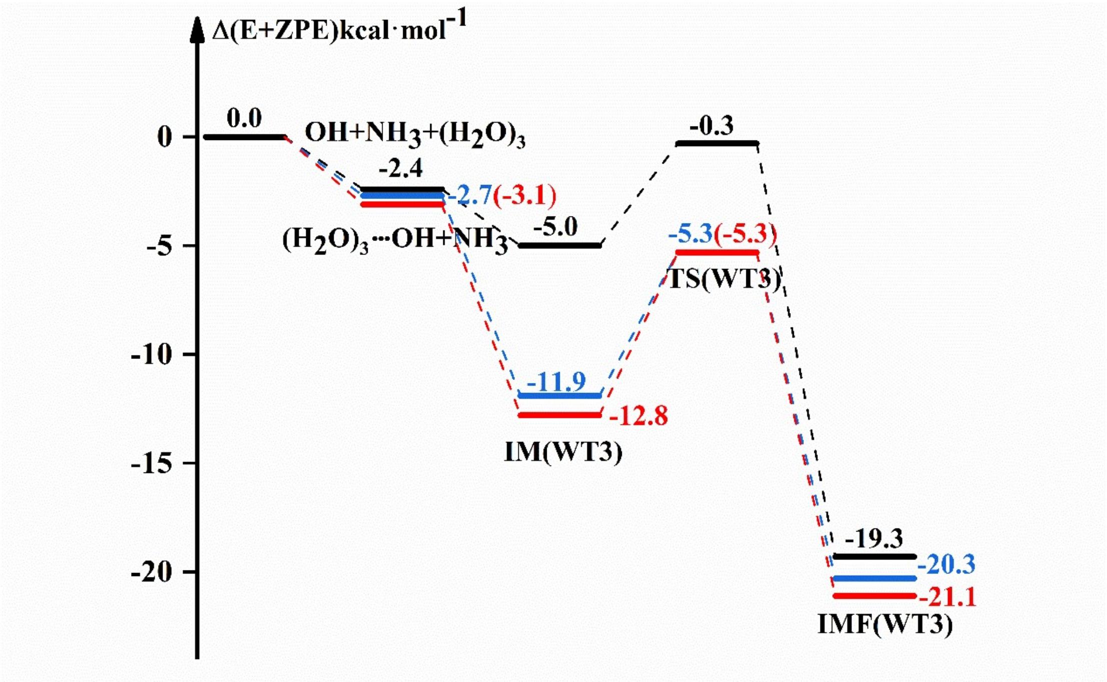

Fig. S15 Schematic energy diagram for the NH_3+OH+$(H_2O)_3$ reaction at the M06-2X/6-311+G(2d,2p) level(blue line) and the CCSD(T)-F12a/cc-pVDZ-F12// M06-2X/6-311+G(2d,2p) level (red line) in the gas phase, and at the M06-2X/6-311+G(2d,2p) level under the implicit solvent model (black line).

Table S12 Relative energy (kcal·mol^{-1}) relative to reactants for $NH_3+OH+(H_2O)_3 \rightarrow NH_2+H_2O+(H_2O)_3$ reaction.

		ΔE_1	ΔE_2	ΔE_3
	DFT(*s*)	-3.6	-5.8	-1.9
WT1	DFT(*g*)	-8.8	-15.8	-9.2
	CCSD(T)(*g*)	-10.6	-16.7	-9.3
	DFT(*s*)	-2.9	-5.3	-1.7
WT2	DFT(*g*)	-2.3	-12.3	-5.6
	CCSD(T)(*g*)	-3.1	-12.8	-5.4
	DFT(*s*)	-2.4	-5.0	-0.3
WT3	DFT(*g*)	-2.7	-11.9	-5.3
	CCSD(T)(*g*)	-3.1	-12.8	-5.3

ΔE_1 is the energy of the bimolecular complex relative to the reactants and ΔE_2 is the energy of the IM relative to the reactants. ΔE_3 is the energy of the TS relative to the reactants.

Table S13 Transition state coordinates of NH_3+OH+$(H_2O)_n$ reaction optimized by M06-2X/6-311+G(2d,2p) in gas and liquid phases, where '*'denotes gas phase conditions.

		Cartesian Coordinate		
TS(WM1)	N	-1.41147300	-0.92891900	-0.10291600

	H	-1.72827200	-1.02104100	0.85919000
	H	-0.47158600	-1.32089200	-0.13452800
	H	-1.30290800	0.15559300	-0.31244900
	O	-0.67416700	1.28401900	0.02125000
	H	0.24477300	0.96059800	0.05762700
	O	1.73722000	-0.25980900	-0.00360000
	H	2.38701400	-0.11520600	-0.69685200
	H	2.24685700	-0.35029400	0.80622200
	N	1.24737300	-1.00235500	-0.10951200
	H	0.29479800	-1.34177200	0.00742100
	H	1.72916100	-1.07303400	0.78215700
	H	1.21380500	0.03097100	-0.44351500
TS(WM2)	O	0.59802600	1.23355200	-0.08764800
	H	0.94199300	1.43512700	0.79367100
	O	-1.77563000	-0.27737600	0.11044000
	H	-1.08718800	0.40338700	0.03445500
	H	-2.40335200	-0.08760600	-0.58994500
	N	-0.26595400	0.79911400	0.03944600
	H	-0.51586500	1.33637800	0.86654100
	H	-0.47927200	1.38446000	-0.76560300
	H	-0.95282900	-0.08573000	0.00071900
TS(WM3)	O	-2.14316100	-0.61781000	0.02487600
	H	-2.71318300	0.01428200	-0.43603400
	O	2.39644400	-0.43386300	0.03236900
	H	2.97451700	0.18169900	-0.42309800
	H	1.52204200	-0.01149400	0.02339800
	N	1.12200200	-1.06965400	-0.10745800
	H	0.13152300	-1.30470400	-0.04723200
	H	1.54669500	-1.21279600	0.80348200
	H	1.20746600	-0.02259000	-0.41696000
TS^*(WM1)	O	0.70056700	1.21496800	-0.08764300
	H	1.03588700	1.40895700	0.79685600
	O	-1.72495900	-0.20599700	0.09436300
	H	-1.08918600	0.51839300	-0.00825800
	H	-2.49126400	0.02855500	-0.42943700
	N	1.12185300	-1.06969600	-0.10743700
	H	0.13136500	-1.30474700	-0.04739900
	H	1.54637500	-1.21284400	0.80358300
	H	1.20740900	-0.02264300	-0.41694300
TS^*(WM2)	O	0.70060700	1.21496800	-0.08762700
	H	1.03606400	1.40895500	0.79682000
	O	-1.72475900	-0.20608400	0.09412900
	H	-1.08904700	0.51840800	-0.00816200
	H	-2.49192400	0.02966900	-0.42786100

TS*(WM3)	N	-0.31674400	0.74716700	0.18438700
	H	-0.58143600	1.12120700	1.09233300
	H	-0.48905500	1.48045500	-0.49979800
	H	-1.02240000	-0.11824200	-0.03219000
	O	-2.20214700	-0.59668900	-0.08859800
	H	-2.69962400	-0.00041000	-0.66379900
	O	2.59209900	0.28895300	-0.29988800
	H	2.89266400	-0.61816700	-0.23694200
	H	1.64938200	0.26276200	-0.09288900
TS(WD1)	N	-1.02668700	-1.57216700	0.21563000
	H	-1.21223200	-1.53348400	1.21474400
	H	-0.04424700	-1.32619700	0.09023100
	H	-1.63479300	-0.77776500	-0.26034000
	O	-1.97825000	0.50845500	-0.33294900
	H	-1.14089100	0.98019000	-0.14756600
	O	0.53558300	1.71090900	0.14989400
	H	1.14154600	0.95467700	0.08530300
	H	0.64776100	2.05871600	1.03791800
	O	1.98840800	-0.67417800	-0.18681300
	H	2.64318900	-0.93193700	0.46839500
	H	2.42055000	-0.78052300	-1.03914100
TS(WD2)	N	0.49464500	0.07360700	0.72833800
	H	-0.13607500	-0.72732900	0.71071900
	H	0.44814800	0.45301400	1.67154400
	H	0.01781800	0.84642700	0.04926600
	O	-1.02367400	1.52173000	-0.28599000
	H	-1.68631900	0.80505400	-0.30653500
	O	-2.38708500	-0.96550300	-0.14321700
	H	-3.15433800	-1.08883400	0.42226900
	H	-2.61037000	-1.39835200	-0.97178900
	O	3.12776800	-0.36115900	-0.43414000
	H	2.25733400	-0.21430800	-0.02523700
	H	3.66521800	-0.75145800	0.25816300
TS(WD3)	N	1.23195900	-1.43580800	-0.11099200
	H	0.21248400	-1.38294400	-0.05189200
	H	1.62162900	-1.70337900	0.78702100
	H	1.66287500	-0.52266600	-0.46944800
	O	1.64363000	0.85906700	-0.06262200
	H	2.03374400	0.85860000	0.82182800
	O	-1.00238200	1.56421000	0.09668600
	H	-0.04209000	1.38479200	0.02483200
	H	-1.22131300	2.13478100	-0.64361300
	O	-1.76950100	-1.07928900	-0.08504700
	H	-1.64693500	-0.11518700	-0.00120400

	H	-2.21807800	-1.35524200	0.71728800
TS*(WD1)	N	0.87105400	-1.07035600	-0.80311900
	H	1.74656600	-1.46205300	-1.14175200
	H	0.63988500	-0.29411200	-1.42285100
	H	1.13117300	-0.57928200	0.20066600
	O	1.61529300	0.34722800	0.90655400
	H	1.05358200	1.07704300	0.58718800
	O	-0.59248500	1.51903500	-0.45239000
	H	-1.18502400	0.82565900	-0.11238000
	H	-1.13156400	2.29374100	-0.61352500
	O	-1.73481800	-0.86796900	0.39267000
	H	-1.69824800	-1.04553600	1.33440300
	H	-0.95767300	-1.30931900	0.01541200
TS*(WD2)	N	0.68335800	-1.22073800	0.68098500
	H	0.53595500	-0.48062500	1.36699500
	H	1.45727200	-1.78279900	1.02758100
	H	1.11265900	-0.68107200	-0.25008000
	O	1.80085000	0.22304700	-0.76468000
	H	1.25623300	0.97789800	-0.47438900
	O	-0.41209000	1.56073200	0.46088600
	H	-0.78070400	2.44460200	0.45713300
	H	-1.08820400	0.97457000	0.07979100
	O	-1.82992300	-0.62139100	-0.51708800
	H	-1.10401300	-1.17200200	-0.18128400
	H	-2.64339800	-1.03451000	-0.22558400
TS*(WD3)	N	1.37379600	-1.31756100	-0.17070700
	H	0.35531600	-1.39333100	-0.09701300
	H	1.81064200	-1.60410800	0.69804300
	H	1.69154800	-0.33616400	-0.47141300
	O	1.52390800	1.00167000	0.00564600
	H	1.90440000	1.02733500	0.89194500
	O	-1.17980100	1.47599100	0.04042500
	H	-0.20508700	1.39441800	0.02524700
	H	-1.41111300	2.03242300	-0.70431000
	O	-1.58497500	-1.23832100	-0.01936400
	H	-1.62291800	-0.26441400	0.00468200
	H	-2.21241200	-1.54795300	0.63411200
TS(WT1)	N	0.36823900	-1.39914300	0.85036700
	H	1.00419200	-1.60595200	0.08308600
	H	-0.56592000	-1.27108000	0.46100600
	H	0.70171700	-0.46735700	1.31981600
	O	1.03463000	0.83002800	1.02206900
	H	0.22834700	1.17090100	0.58055100
	O	-1.34465000	1.65574700	-0.22363300

	H	-1.83833900	0.82780800	-0.34296300
	H	-1.22090300	2.01469400	-1.10575700
	O	-2.51579900	-0.89517800	-0.29976500
	H	-2.73342800	-1.29078300	-1.14874300
	H	-3.28697700	-1.03668700	0.25686500
	O	2.67945300	-0.20947300	-0.99016700
	H	2.20510000	0.28605400	-0.29970600
	H	3.61096600	-0.04045200	-0.83523500
TS(WT2)	N	-0.34815800	-1.46933500	0.75388400
	H	0.56502000	-1.40690200	0.29833400
	H	-1.06536000	-1.58428200	0.04144700
	H	-0.55635900	-0.55779000	1.30939300
	O	-0.74998300	0.80017800	1.04373800
	H	-1.47422600	0.73421500	0.39429200
	O	2.43987800	-1.02163800	-0.43118600
	H	2.55039300	-1.26261500	-1.35344300
	H	2.24266800	-0.06659600	-0.42948700
	O	-2.73668700	-0.04346400	-0.82258100
	H	-3.65717900	0.02724900	-0.55523400
	H	-2.70841200	0.29022600	-1.72345000
	O	1.59669500	1.59497500	-0.11536200
	H	1.42381200	2.09529300	-0.91620100
	H	0.71752000	1.37614700	0.26029500
TS(WT3)	N	-0.85422400	0.01654000	1.63719900
	H	-1.56036600	-0.66716000	1.37142100
	H	-1.20425500	0.94706000	1.41829800
	H	0.04781000	-0.16432000	1.05888300
	O	0.37877300	-0.20366000	-0.30789500
	H	0.07310000	0.68037600	-0.58573600
	O	-1.81157700	-1.94164700	-0.59171200
	H	-0.98925400	-1.43011300	-0.66845100
	H	-1.59932600	-2.82295400	-0.90555400
	O	-0.83252800	2.35687500	-0.48724800
	H	-0.30869300	3.15684000	-0.58365200
	H	-1.57912800	2.46878200	-1.08214900
	O	3.19152000	-0.31884700	0.01370200
	H	2.22768600	-0.28620000	-0.09632300
	H	3.46249500	-1.13985200	-0.40191700
TS^{*}(WT1)	N	0.08902900	-1.19681400	1.50148700
	H	-0.62693700	-0.47870500	1.58555800
	H	0.98241800	-0.72133800	1.38986200
	H	-0.10243400	-1.79109600	0.59353500
	O	-0.24123900	-1.59920300	-0.75005900
	H	0.59349800	-1.14408500	-0.96319000

	O	2.00803000	0.14404800	-0.57326600
	H	1.48173400	0.92129400	-0.31640200
	H	2.73483300	0.46140900	-1.10970000
	O	0.12548800	1.88550200	0.40324200
	H	-0.72064500	1.53616100	0.06256400
	H	0.00737600	2.82453500	0.54713700
	O	-1.99432100	0.40196400	-0.40352500
	H	-1.45443500	-0.37734400	-0.66883500
	H	-2.70226700	0.48837900	-1.04206600
TS*(WT2)	N	-0.67491100	-0.64196900	1.30791300
	H	-0.10069700	-1.39630900	0.93612500
	H	-1.65810500	-0.81661200	1.10522800
	H	-0.38331300	0.28593200	0.82787500
	O	-0.32427200	0.80782900	-0.48141100
	H	0.04281000	0.06089900	-0.98292800
	O	1.54219500	-1.40983600	-0.57581600
	H	2.09984200	-1.98290000	-1.10314800
	H	2.10132000	-0.67358000	-0.27569300
	O	-3.04537700	0.10581900	-0.32284800
	H	-2.23043600	0.56337300	-0.57940900
	H	-3.76751300	0.70475200	-0.51249800
	O	2.39617500	1.11542300	0.17663700
	H	2.60530100	1.45733600	1.04689000
	H	1.46538700	1.33700800	0.00966900
TS*(WT3)	N	-0.70691900	0.70749700	1.28542100
	H	-1.67761400	0.88747700	1.03299500
	H	-0.10020700	1.43466500	0.91079700
	H	-0.40480000	-0.25222400	0.86888400
	O	-0.31063100	-0.83294000	-0.40621200
	H	0.03394900	-0.10665900	-0.95042800
	O	3.03364200	-0.13604900	-0.36439900
	H	-2.20994100	-0.61101300	-0.55437600
	H	-3.74418400	-0.76912400	-0.46717700
	O	1.53606900	1.39742100	-0.62093300
	H	2.11536400	0.67751100	-0.32108200
	H	2.09288200	2.03075200	-1.07484400
	O	2.36653500	-1.07016200	0.31917200
	H	1.45212100	-1.31294900	0.09537400
	H	2.92421800	-1.79707200	0.04088300
$TS[(H_2O)_5]$	O	1.59176600	-0.01753400	0.57761600
	H	1.01010000	-0.73996800	0.25482300
	O	-0.38658800	-1.93940900	0.15832200
	H	-1.03454600	-1.66187200	-0.50531600
	H	-0.69825900	-1.54516700	0.98980800

	O	-2.37280500	-0.81324200	-1.59391400
	H	-3.24480600	-1.21908100	-1.59253200
	H	-2.16297300	-0.65087000	-2.51840500
	O	-0.80784400	-0.16536800	2.32326000
	H	0.12893500	0.05653500	2.35214000
	H	-1.18522200	0.44431900	1.65825700
	O	0.66593900	2.29303400	-0.60734500
	H	0.82682000	2.30047400	-1.55388000
	H	1.09515100	1.48302000	-0.26811700
	O	-1.82279000	1.35264300	0.23852400
	H	-1.02360300	1.76471100	-0.13603400
	H	-2.09412600	0.68885000	-0.41152800
	N	3.44702900	-0.67660000	-0.68268100
	H	3.37086900	0.05853700	-1.37956800
	H	3.05899100	-1.52688600	-1.08161600
	H	2.88204500	-0.39738800	0.19904200
TS[$(H_2O)_6$]	O	-1.87110900	-2.31494600	-0.77937400
	H	-2.24472300	-1.42946500	-0.95218500
	H	-2.58468300	-2.83654800	-0.40475600
	O	-0.07499100	-1.01402000	0.85981900
	H	-0.58385300	-1.68479200	0.36996100
	H	0.83507900	-1.34260700	0.99104000
	O	-1.84839700	0.92179100	1.61296800
	H	-1.23741500	0.16458600	1.63538100
	H	-2.37723800	0.88100000	2.41328200
	O	3.05793500	0.27446500	-1.03035900
	H	2.14893900	0.59730600	-1.17715400
	H	3.58170300	1.04936600	-0.81242200
	O	-2.51483200	0.37071100	-1.09096600
	H	-2.51317800	0.62759600	-0.15467200
	H	-1.64838400	0.66641700	-1.40755800
	O	2.61953300	-1.51851400	1.03324900
	H	2.88464600	-0.92306900	0.30592000
	H	2.95335500	-2.38761400	0.79852100
	O	0.33467300	0.87130200	-1.17197700
	H	0.19645800	0.24005000	-0.43122200
	N	0.28085500	2.66802700	0.32800500
	H	-0.42684400	2.27702600	0.95109600
	H	1.19815700	2.52710800	0.74155600
	H	0.22949800	2.17114000	-0.62970100
TS[$(H_2O)_7$]	O	2.92675800	-0.37776600	0.52620000
	H	2.64052900	0.46148900	0.11000000
	O	-2.40818600	1.53958400	-0.27813800
	H	-2.86157500	0.70170100	-0.11070800

	H	-1.73801100	1.62207200	0.42544800
	O	-3.47053200	-1.14750100	0.01549700
	H	-4.01480100	-1.44816300	-0.71851000
	H	-3.95238300	-1.39057500	0.81171800
	O	1.66878400	1.82922400	-0.64841000
	H	0.97739600	1.39270200	-1.19832800
	H	2.05760700	2.53015200	-1.17709700
	O	0.74421400	-0.78900000	2.06063600
	H	0.90680100	-1.23228900	2.89667800
	H	1.60931200	-0.70572500	1.59841200
	O	-0.67479100	-1.72428500	-0.13023700
	H	-0.31255000	-1.46581500	0.73513700
	H	-1.63901300	-1.67130500	-0.04728500
	O	-0.24169500	1.80616100	1.46996400
	H	0.45528200	2.01614000	0.82971100
	H	0.01651100	0.94150500	1.82598900
	O	-0.33264900	0.48046900	-1.82774400
	H	-1.10368900	0.98055400	-1.50309200
	H	-0.37786200	-0.33378300	-1.29266600
	N	2.20476600	-1.61339200	-1.32378700
	H	2.86504700	-1.29452300	-0.52246900
	H	1.30766300	-1.91315600	-0.94550100
	H	2.03514800	-0.83232600	-1.95306900
TS[$(H_2O)_8$]	O	2.92675800	-0.37776600	0.52620000
	H	2.64052900	0.46148900	0.11000000
	O	-2.40818600	1.53958400	-0.27813800
	H	-2.86157500	0.70170100	-0.11070800
	H	-1.73801100	1.62207200	0.42544800
	O	-3.47053200	-1.14750100	0.01549700
	H	-4.01480100	-1.44816300	-0.71851000
	H	-3.95238300	-1.39057500	0.81171800
	O	1.66878400	1.82922400	-0.64841000
	H	0.97739600	1.39270200	-1.19832800
	H	2.05760700	2.53015200	-1.17709700
	O	0.74421400	-0.78900000	2.06063600
	H	0.90680100	-1.23228900	2.89667800
	H	1.60931200	-0.70572500	1.59841200
	O	-0.67479100	-1.72428500	-0.13023700
	H	-0.31255000	-1.46581500	0.73513700
	H	-1.63901300	-1.67130500	-0.04728500
	O	-0.24169500	1.80616100	1.46996400
	H	0.45528200	2.01614000	0.82971100
	H	0.01651100	0.94150500	1.82598900
	O	-0.33264900	0.48046900	-1.82774400

	H	-1.10368900	0.98055400	-1.50309200
	H	-0.37786200	-0.33378300	-1.29266600
	N	2.20476600	-1.61339200	-1.32378700
	H	2.86504700	-1.29452300	-0.52246900
	H	1.30766300	-1.91315600	-0.94550100
	H	2.03514800	-0.83232600	-1.95306900
TS[$(H_2O)_{10}$]	O	2.08068600	1.68748700	0.96774500
	H	1.88315000	2.15727700	-0.81104300
	H	2.57946900	0.85959100	1.05257700
	O	3.00235800	-0.89172600	0.43781300
	H	3.88425500	-1.26896300	0.48837900
	H	2.75324200	-0.86831500	-0.51455800
	O	-0.38369100	-2.31901200	-1.53211700
	H	-1.20988400	-1.91266800	-1.19103400
	H	-0.64391700	-3.11324900	-2.00517300
	O	1.95152400	-0.70808600	-2.05305100
	H	1.75660900	0.24369200	-2.07035100
	H	1.09273100	-1.14356500	-1.92313700
	O	-1.26263600	-1.00305500	2.20291100
	H	-0.50273300	-1.50457700	1.82868600
	H	-1.55275200	-1.49209700	2.97753000
	O	-2.66149900	-1.38376800	-0.29891800
	H	-3.05517700	-0.56302300	-0.64164100
	H	-2.26258800	-1.14800200	0.55418900
	O	-0.23662800	1.58279700	2.49722800
	H	1.40615900	1.69821000	1.67029600
	H	-0.60937200	0.68285200	2.41758100
	O	0.73162600	-2.47357200	1.06125900
	H	1.56031400	-1.97293600	0.99108500
	H	0.39446200	-2.51315000	0.14937700
	O	-3.68626800	1.06484700	-1.26746300
	H	-0.30713300	1.81800700	3.42569000
	H	-4.46518200	1.38177300	-0.80380800
	O	1.61805200	2.12917500	-1.74666900
	H	2.23741700	2.69242900	-2.21763000
	H	-2.94796700	1.62792500	-0.96714500
	H	0.69727100	0.56540100	0.13454400
	O	-0.10811600	0.10966700	-0.17091600
	N	-1.21945000	2.17457700	-0.36865600
	H	-0.94588200	1.08990900	-0.37520900
	H	-1.04741900	2.47854400	0.58983600
	H	-0.52218700	2.62487100	-0.96101600
TS[$(H_2O)_{11}$]	O	2.08068600	1.68748700	0.96774500
	H	1.88315000	2.15727700	-0.81104300

	H	2.57946900	0.85959100	1.05257700
	O	3.00235800	-0.89172600	0.43781300
	H	3.88425500	-1.26896300	0.48837900
	H	2.75324200	-0.86831500	-0.51455800
	O	-0.38369100	-2.31901200	-1.53211700
	H	-1.20988400	-1.91266800	-1.19103400
	H	-0.64391700	-3.11324900	-2.00517300
	O	1.95152400	-0.70808600	-2.05305100
	H	1.75660900	0.24369200	-2.07035100
	H	1.09273100	-1.14356500	-1.92313700
	O	-1.26263600	-1.00305500	2.20291100
	H	-0.50273300	-1.50457700	1.82868600
	H	-1.55275200	-1.49209700	2.97753000
	O	-2.66149900	-1.38376800	-0.29891800
	H	-3.05517700	-0.56302300	-0.64164100
	H	-2.26258800	-1.14800200	0.55418900
	O	-0.23662800	1.58279700	2.49722800
	H	1.40615900	1.69821000	1.67029600
	H	-0.60937200	0.68285200	2.41758100
	O	0.73162600	-2.47357200	1.06125900
	H	1.56031400	-1.97293600	0.99108500
	H	0.39446200	-2.51315000	0.14937700
	O	-3.68626800	1.06484700	-1.26746300
	H	-0.30713300	1.81800700	3.42569000
	H	-4.46518200	1.38177300	-0.80380800
	O	1.61805200	2.12917500	-1.74666900
	H	2.23741700	2.69242900	-2.21763000
	H	-2.94796700	1.62792500	-0.96714500
	H	0.69727100	0.56540100	0.13454400
	O	-0.10811600	0.10966700	-0.17091600
	N	-1.21945000	2.17457700	-0.36865600
	H	-0.94588200	1.08990900	-0.37520900
	H	-1.04741900	2.47854400	0.58983600
	H	-0.52218700	2.62487100	-0.96101600
$TS[(H_2O)_{13}]$	O	0.71451300	0.26709900	-1.25245100
	O	-1.66754400	-0.16936200	-2.65493800
	O	2.88876300	-2.41072300	-0.32738000
	O	2.11820500	-0.59872300	1.66083500
	O	3.46791400	-0.08037100	-1.63613900
	O	-0.61371600	2.23755100	0.27700500
	O	0.17103500	-2.47280700	-1.06551200
	O	-1.94223800	1.18883300	2.53624900
	O	1.70742400	3.70861800	0.16050300
	O	-3.70376500	0.22727100	0.52423800

O	-2.75806700	1.98172200	-1.47910300
O	-2.63267400	-2.00304600	-0.70182900
O	3.36264900	1.54059800	0.64797600
H	0.42464200	0.92098400	-0.59464700
H	1.66134400	0.37874800	-1.43027600
H	-2.01329200	-0.89069200	-2.10232200
H	-0.75559000	-0.03654200	-2.34759900
H	2.68102800	-1.89402700	0.47076100
H	2.02249100	-2.71023100	-0.64119600
H	2.54896800	0.23822100	1.36140100
H	2.35573300	-0.71491000	2.58486600
H	3.37279400	-0.99758000	-1.30316200
H	3.93842500	-0.12268300	-2.47173300
H	-1.35413800	2.31536400	-0.35118700
H	-1.01625200	2.01144700	1.13172900
H	-0.75990300	-2.57094200	-0.81956800
H	0.32315300	-1.51438000	-0.99122200
H	-1.42929900	0.36653100	2.39580600
H	-1.93765800	1.38368000	3.47614600
H	1.75374800	4.42559500	0.79736200
H	0.81553600	3.32566600	0.24790800
H	-3.54432200	0.90219100	-0.15455900
H	-3.25678300	0.56178100	1.31577000
H	-3.19086900	2.61949900	-2.05137000
H	-2.41627400	1.25270400	-2.04655500
H	-3.40013500	-2.53934400	-0.91658600
H	-2.96181800	-1.26595100	-0.14598400
H	3.59038500	1.16652300	-0.21627400
H	2.81827300	2.33175100	0.47563100
H	0.31649400	-0.68691300	1.37073700
O	-0.65836300	-0.77151900	1.30646500
N	-0.49303800	-3.02844000	1.90317800
H	-0.11019100	-2.89228600	2.83507100
H	-0.93695000	-2.08968800	1.55872100
H	0.27863500	-3.23658800	1.27272500

Table S14 Calculated electronic energies, zero-point energies and free energies calculated by M06-2X/6-311+G(2d,2p), where '*' indicates the gas phase condition. Note that the liquid phase free energy refers to the solute free energy.

	E(Hartree)	ZPE(Hartree)	G(Hartree)
NH_3	-56.5545799	0.034533	-56.5380719
OH	-75.7331858	0.008536	-75.7415718
H_2O	-76.4311385	0.021552	-76.4278795
NH_3^*	-56.5492533	0.034642	-56.5326353

OH^*	-75.7283893	0.008636	-75.7366723
H_2O^*	-76.424065	0.021684	-76.420013
TS	-132.2821155	0.042313	-132.2648945
TS^*	-132.2732869	0.042328	-132.2557119
TS(WM1)	-208.7208125	0.066846	-208.6849075
TS(WM2)	-208.7216075	0.068521	-208.6828455
TS(WM3)	-208.7194479	0.066283	-208.6842149
TS^*(WM1)	-208.7117107	0.068842	-208.6723447
TS^*(WM2)	-208.7117107	0.068838	-208.6723557
TS^*(WM3)	-208.7048494	0.066157	-208.6711774
TS(WD1)	-285.1619712	0.091869	-285.1054722
TS(WD2)	-285.1588068	0.091357	-285.1036368
TS(WD3)	-285.1642257	0.094239	-285.1037677
TS^*(WD1)	-285.1513618	0.093516	-285.0909568
TS^*(WD2)	-285.1503315	0.093517	-285.0897965
TS^*(WD3)	-285.1520698	0.09482	-285.0911818
TS(WT1)	-361.602119	0.117339	-361.52491
TS(WT2)	-361.6030589	0.118605	-361.5233529
TS(WT3)	-361.5987427	0.11765	-361.5224007
TS^*(WT1)	-361.5961474	0.120155	-361.5134084
TS^*(WT2)	-361.5899549	0.120335	-361.5075769
TS^*(WT3)	-361.5895586	0.119912	-361.5082176
$TS[(H_2O)_5]$	-514.4859031	0.168301	-514.3573993
$TS[(H_2O)_6]$	-590.9361145	0.195521	-590.7817334
$TS[(H_2O)_7]$	-667.381713	0.220951	-667.2048861
$TS[(H_2O)_8]$	-743.822342	0.24727	-743.620445
$TS[(H_2O)_{10}]$	-896.7108485	0.29622	-896.4636538
$TS[(H_2O)_{11}]$	-973.1520175	0.321353	-972.8830378
$TS[(H_2O)_{13}]$	-1126.042203	0.373276	-1125.728756
